# Was the evolution of faster stomata driven by increased gas exchange rates rather than increasing water use efficiency?

**DOI:** 10.1111/nph.70830

**Published:** 2025-12-24

**Authors:** Robert A. Brench, Matthew J. Wilson, Sarah J. Thorne, Andrew J. Fleming, Julie E. Gray

**Affiliations:** ^1^ Plants, Photosynthesis and Soils, School of Biosciences University of Sheffield Western Bank Sheffield S10 2TN UK

**Keywords:** evolution, light, photosynthesis, stomata, water

## Abstract

- Following changes in light flux, photosynthesis (*A*) typically adjusts more quickly than stomatal conductance (*g*_s_), which is dependent on changes in stomatal aperture. Faster stomatal responses are proposed to reduce water loss and enhance growth in dynamic light environments.
- Stomatal opening and closing parameters were determined in a range of species across the land plant phylogeny using infrared gas exchange analysis to monitor *A* and *g*_s_, following step changes in light flux.
- The acquisition of abaxial stomata and dumbbell‐shaped guard cells in angiosperms coincides with two distinct increases in photosynthetic capacity*.* Species with dumbbell‐shaped guard cells achieved larger changes in *A* and faster maximum rates of *g*_s_ adjustment than species with kidney‐shaped guard cells. However, species with dumbbell‐shaped guard cells did not open or close their stomata in a significantly shorter time once opening began, nor did they achieve higher water use efficiency.
- Surprisingly, there were no strong correlations between stomatal size and speed parameters and no differences in biomass accumulation or water use between plants grown under constant or fluctuating light. We therefore propose increased gas‐exchange rates, rather than faster stomatal response times, as the evolutionary driver for the acquisition of dumbbell‐shaped guard cells.

## Introduction

Stomata, epidermal pores that control the uptake of CO_2_ into, and water vapour loss out of, leaves (Cowan, 1978), consist of two guard cells. Changes in guard cell turgor adjust pore apertures. The maximum level of stomatal conductance (*g*_s_) is dependent on the size of the stomatal pore and the density of stomata (Franks *et al*., 2009), and steady state values of stomatal conductance and carbon assimilation (*A*) are often tightly correlated (Wong *et al*., 1979; Farquhar & Sharkey, 1982).

Plants respond to constantly changing environmental conditions through opening and closing stomata (Pearcy, 1990; Vialet‐Chabrand *et al*., 2017). Amongst the most dynamic environmental signals that affect stomatal apertures is a change in light level (photosynthetic photon flux density, or PPFD). High PPFD drives an increase in guard cell turgor, which opens stomata to support increased photosynthesis, whereas low PPFD causes stomatal closure, which reduces water loss. PPFD can fluctuate on scales of seconds to seasons (Pearcy, 1990; Slattery *et al*., 2018; Long *et al*., 2022), and under such dynamic conditions, there is a temporary disconnect between *A* and *g*_s_ for perhaps several minutes whilst pore apertures adjust. Slow stomatal opening during increasing irradiance can limit photosynthesis, as adjustments in *A* are often an order of magnitude quicker than adjustments in *g*_s_ (Knapp & Smith, 1987, 1989; Vico *et al*., 2011; McAusland *et al*., 2016; Zhang *et al*., 2022). This slower response of *g*_*s*_ to changing light conditions is regarded as a source of inefficiency in plants (Lawson & Blatt, 2014; Faralli *et al*., 2019; Lawson & Vialet‐Chabrand, 2019). Conversely, slow stomatal closure during decreasing irradiance results in superfluous water loss for very little carbon gain, which transiently decreases water use efficiency (WUE; Lawson & Blatt, 2014; McAusland *et al*., 2016; Lawson & Vialet‐Chabrand, 2019; Eyland *et al*., 2021).

Slow stomatal pore adjustment has been proposed to limit *A* by as much as 20% in fluctuating light environments, imposing a significant loss to potential crop yields (Lawson & Blatt, 2014). It is therefore assumed that crops with faster stomatal responses would be more efficient in dynamic light environments, and several studies support this (Farquhar & Sharkey, 1982; Knapp & Smith, 1987; Grantz & Assmann, 1991; McAusland *et al*., 2016; Matthews *et al*., 2018; Lawson & Vialet‐Chabrand, 2019).

Various stomatal traits have been proposed to facilitate faster aperture response speeds. These include small stomatal size, geometric innovations such as the evolution of dumbbell‐shaped guard cells and the presence of subsidiary cells. Smaller stomata are expected to respond more rapidly to changes in the external environment due to their greater relative surface area to volume ratio (Hetherington & Woodward, 2003; Franks *et al*., 2009; Drake *et al*., 2013; Kardiman & Ræbild, 2018; Israel *et al*., 2022; Ozeki *et al*., 2022; Zhang *et al*., 2022). Assuming that the density and activity of ion transporters in the guard cell membrane that facilitate turgor change are similar, smaller cells should theoretically be able to adjust turgor more quickly than larger ones (Lawson & Blatt, 2014; Raven, 2014). Some evidence exists supporting this hypothesis, particularly among closely related species that share an ecological niche (Drake *et al*., 2013; Kardiman & Ræbild, 2018; Ozeki *et al*., 2022; Zhang *et al*., 2022); however, the role of stomatal size appears to be less consistent in influencing stomatal response speeds across more distantly related species (Elliott‐Kingston *et al*., 2016; McAusland *et al*., 2016; Deans *et al*., 2019).

The lack of consistent associations between stomatal size and speed suggests that other factors influence turgor pressure and aperture response speeds. The density of guard cell ion transporters has been shown to vary between species, which has implications for the ability to modulate turgor and hence pore width (Blatt, 1988; Blatt & Gradmann, 1997). Some stomatal morphologies can be distinguished by the presence of dedicated subsidiary cells surrounding the guard cells. These have been hypothesised to enable the provision of ions to facilitate rapid shifts in cell turgor pressure, and the consequent reciprocal change in turgor between guard and subsidiary cells has been suggested to decrease the mechanical advantage of the epidermis (Willmer & Pallas, 1973; Franks & Farquhar, 2007; Gray *et al*., 2020; Durney *et al*., 2023; Jaafar & Anderson, 2024). Whilst subsidiary cells are not ubiquitous across land plants, they can be found across a range of taxa.

One key innovation associated with increased stomatal speeds is the dumbbell‐shaped guard cell morphology found in some monocot clades, including the grasses. These elongated guard cells have distinct bulbous ends joined by a stiffened rod region and are symplastically connected (Nunes *et al*., 2020; Durney *et al*., 2023; Wilson *et al*., 2025). Compared to species with kidney‐shaped guard cells, plants with dumbbell‐shaped guard cells open and close their stomata more quickly and this innovation in cellular morphology is believed to have aided in the colonisation and success of grasses in more arid conditions (Hetherington & Woodward, 2003; Vico *et al*., 2011; McAusland *et al*., 2016). Dumbbell‐shaped guard cells are always accompanied by flanking subsidiary cells that can contribute to stomatal response speed. Indeed, a *Brachypodium distachyon* mutant, *bdmute*, which lacks lateral subsidiary cells, whilst retaining dumbbell‐shaped guard cells, opens and closes its stomata more slowly in response to light (Raissig *et al*., 2017; Nguyen & Blatt, 2024).

Ferns and lycophytes are often considered to have slow or less environmentally sensitive stomata, with some authors suggesting that species of earlier diverging clades lack the ability to respond to changing environmental stimuli through the active movement of ions across the guard cell membrane (Brodribb & McAdam, 2011; McAdam & Brodribb, 2012). However, recent studies show that some ferns are able to rapidly open stomata in response to light (Xiong *et al*., 2018; Deans *et al*., 2019; Cai *et al*., 2021) and that rates of opening but not closing are negatively correlated with stomatal size (Kübarsepp *et al*., 2019). Experiments with mosses, lycophytes and ferns indicate that active adjustment of stomatal apertures arose very early in land plant evolution (Ruszala *et al*., 2011, Chater *et al*., 2016; Clark *et al*., 2022; Ηorak *et al*., 2017; Plackett *et al*., 2021).

Links between photosynthetic type and stomatal response speeds have been suggested (Ozeki *et al*., 2022; Rui *et al*., 2024). Most plant species operate with a C_3_ photosynthetic mechanism (Sage, 2004). This is susceptible to high levels of photorespiration through the oxygenase activity of Rubisco, which decreases photosynthetic efficiency. An alternative C_4_ photosynthetic mechanism has evolved in some species. This produces a four‐carbon compound, enabling the release of carbon dioxide near the site of fixation, increasing the rate of carboxylation relative to oxygenation, and enhancing photosynthetic efficiency (Sage *et al*., 2012). The C_4_ mechanism has arisen multiple times and is thought to be an adaptation to limit water loss by maintaining carbon fixation at low levels of stomatal conductance, thereby increasing WUE (Schulze *et al*., 1996). C_4_ photosynthesis is often associated with more rapid stomatal closure responses to achieve reductions in water loss (Ozeki *et al*., 2022).

There is much interest in engineering more rapid stomatal responses with the aim of increasing WUE. For example, increases in stomatal response rate have been achieved through the introduction of a synthetic, light‐gated K^+^ channel into *Arabidopsis thaliana* guard cells. This resulted in more rapid stomatal opening and closure, and increased biomass accumulation under fluctuating light (Papanatsiou *et al*., 2019). However, the steady state *g*_s_ achieved during stomatal opening was also enhanced, making it difficult to isolate the impact on response rate alone.

**Fig. 1:**
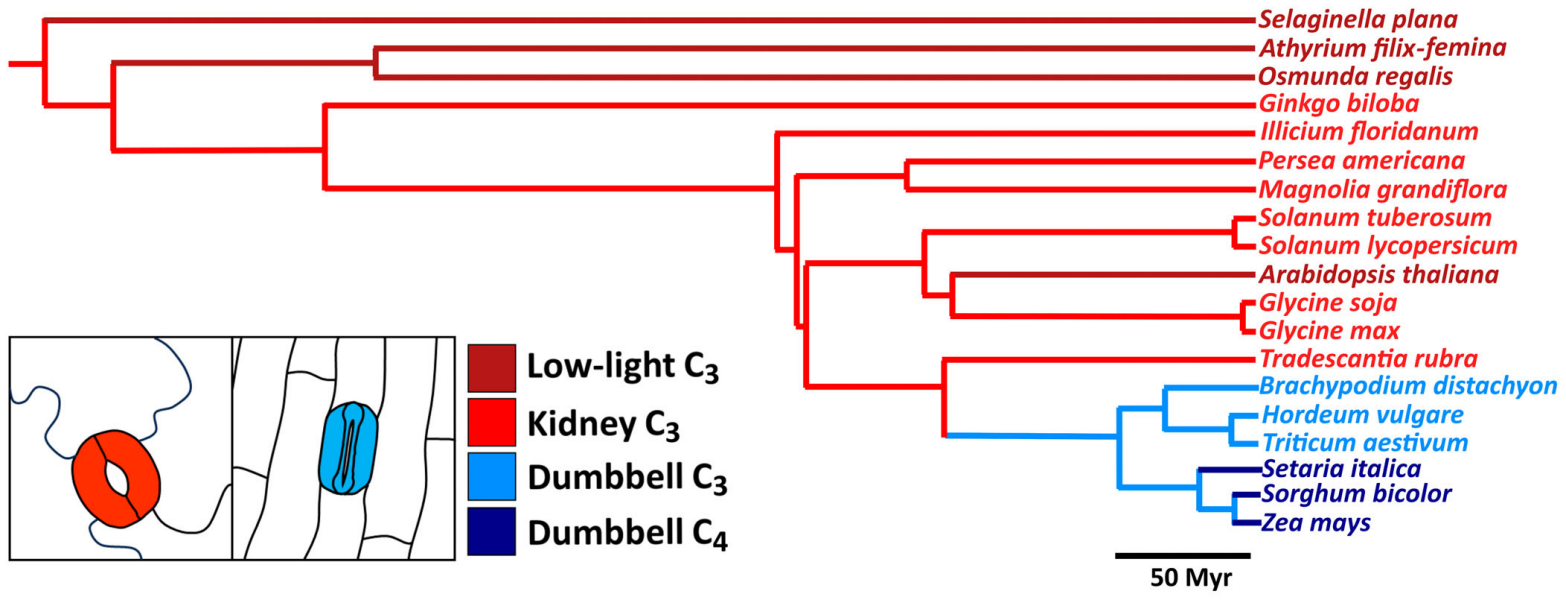
Phylogenetic tree of species used in this study. Bottom left inset shows typical morphologies of stomata with kidney or dumbbell‐shaped guard cells. Line and cell colours indicate species groups: dark red, kidney (low light) C_3_; light red, kidney (high light) C_3_; light blue, dumbbell C_3_; dark blue, dumbbell C_4_.

Here, the potential advantages of rapid stomatal dynamics were explored by comparing the light‐induced speeds of adjustment of stomatal conductance, photosynthesis and WUE in species spanning several land plant evolutionary clades, sharing a most recent common ancestor > 400 million years ago (Ma) (Magallón *et al*., 2013) (Fig. 1a). Species were sampled across the diversity of vascular plants, including a lycophyte and two ferns, a gymnosperm, several angiosperms (encompassing early angiosperm species through to later diverging monocot grasses), three species with C_4_ photosynthetic biochemistry, some low‐light adapted plants, examples of plants with stomata on either one or both sides of the leaf (i.e. hypostomatous or amphistomatous), and several species that have been domesticated as crop plants (see Table 1). To investigate whether rapid stomatal responses generally confer an advantage, biomass gain, water loss and WUE were compared for six species following growth under either constant or fluctuating light conditions.

**Table 1:** Summary of species used and their phylogenetic groups.

Species	Clade	Family	Notes
*Selaginella plana* (1)	Lycophyte	Selaginellaceae	Tropical lycophyte
*Athyrium filix‐femina* (2)	Fern	Athyriaceae	Temperate fern
*Osmunda regalis* (3)	Fern	Osmundaceae	Temperate fern
*Ginkgo biloba* (4)	Gymnosperm	Ginkgoaceae	Tree
*Illicium floridanum* (5)	Angiosperm	Schisandraceae	Shrub
*Persea americana* (6)	Angiosperm	Lauraceae	Tree/crop
*Magnolia grandiflora* (7)	Angiosperm	Magnoliaceae	Tree
*Solanum tuberosum* (8)	Angiosperm	Solanaceae	Crop
*Solanum lycopersicum* (9)	Angiosperm	Solanaceae	Crop
*Arabidopsis thaliana* (10)	Angiosperm	Brassicasceae	Model organism
*Glycine soja* (11)	Angiosperm	Fabaceae	Wild progenitor
*Glycine max* (12)	Angiosperm	Fabaceae	Crop
*Tradescantia rubra* (13)	Angiosperm	Commelinaceae	Nongrass
*Brachypodium distachyon* (14)	Angiosperm	Poaceae	Wild grass
*Hordeum spontaneum* (15)	Angiosperm	Poaceae	Wild progenitor
*Hordeum vulgare* (16)	Angiosperm	Poaceae	Crop
*Triticum boeoticum* (17)	Angiosperm	Poaceae	Wild progenitor
*Triticum araraticum* (18)	Angiosperm	Poaceae	Wild progenitor
*Triticum durum* (19)	Angiosperm	Poaceae	Crop
*Triticum aestivum* (20)	Angiosperm	Poaceae	Crop
*Zea mays* (21)	Angiosperm	Poaceae	C_4_ crop
*Sorghum bicolor* (22)	Angiosperm	Poaceae	C_4_ crop
*Setaria italica* (23)	Angiosperm	Poaceae	C_4_ crop

Colour coding of species groups: dark red, kidney (low light) C_3_; light red, kidney (high light) C_3_; light blue, dumbbell C_3_; dark blue, dumbbell C_4_.

## Materials and Methods

### Plant material and growth conditions

Plants were propagated from seed (or tubers for *Solanum tuberosum L.*), except for *Selaginella plana (Desv. ex Poir.)*, *Athyrium filix‐femina (L.) Roth.*, *Osmunda regalis L., Illicium floridanum J. Ellis, Ginkgo biloba* L. and *Magnolia grandiflora L.*, which were obtained as plants, and measurements were taken from leaves that had developed under controlled growth environment conditions. *Triticum aestivum* L. (cv Cougar), *Triticum durum* Desf. (cv Voilur), *Tradescantia Andersoniana Group 'Rubra', Brachypodium distachyon* L. (line Bd21‐3), *Hordeum vulgare* L. (cv Golden Promise), *Hordeum spontaneum K. Koch., Triticum boeoticum* Boiss. (line 18 344), *Triticum araraticum* Boiss. (line 18 358), *Arabidopsis thaliana* (L.) Heynh. (Col‐0), *Zea mays L., Setaria italica* (L.) P. Beauv. and *Sorghum bicolor* (L.) Moench were grown in Conviron PGR15c controlled growth chambers (Conviron, Canada); *Solanum tuberosum* (King Edward), *Solanum lycopersicum* L. (Moneymaker), *Glycine max* (L.) Merr. (Williams 82) and *Glycine soja* Siebold & Zucc. were grown in glasshouses (with controlled environment temperature humidity and light regulation); *A. filix‐femina*, *O. regalis, I. floridanum, G. biloba* and *M. grandiflora* were grown in an air‐conditioned, temperature‐controlled room under a LED light source (AP673L, Valoya, Finland). For further details, see Supporting Information Table S1.

### Phylogeny

TimeTree (Kumar *et al*., 2022) was used to estimate the time periods separating the most recent common ancestors of species used in this study, and a phylogeny was created to illustrate evolutionary relationships (as described by Clarke *et al*., 2011).

### Size and density measurements

Abaxial and adaxial leaf epidermal impressions were taken using dental resin (Coltene Whaledent, Switzerland) from the central region of mature leaves, avoiding major veins. Where possible, impressions were taken from the same location on the leaf where gas exchange experiments had previously been conducted (*n* = 3–8 leaves per species). Nail varnish was applied to the resin impressions and allowed to dry before slide mounting and imaging using light microscopy (n300‐M; Brunel, London, UK). Four fields of view (9.1–0.29 mm^2^) were analysed per leaf impression to ensure that results were representative. The number of stomata was counted and scaled to calculate density per 1 mm^2^. The stomatal ratio was calculated by dividing the mean adaxial density by the mean abaxial density. The length and width of guard cell pairs were measured from two stomatal complexes per field of view (i.e. eight per leaf surface on at least three leaves) using imagej (Schindelin *et al*., 2012). Stomatal complex size was calculated using the equation for an ellipse.

### Infrared gas analysis

Infrared gas analysis (IRGA) was carried out to analyse carbon assimilation (*A*) and stomatal conductance (*g*_s_) before, during, and after an increase in PPFD using a LI‐COR 6800 portable photosynthesis system as a light source (fitted with 6800‐01A multiphase flash fluorometer; LI‐COR Biosciences, Lincoln, NE, USA). The IRGA chamber was clipped onto a mature leaf, which remained attached to the plant within the controlled growth environment. Leaves were acclimated inside the chamber, with the light source on the adaxial surface, at an appropriate low PPFD for the species, until steady‐state conductance was reached (*c*. 20–60 min). After 5 min at steady state, PPFD was increased to an appropriate high level for the species for 90 min and then reduced again to low PPFD for 90 min. Data were logged every minute (except for *S. italica*, which was logged every 10 s due to its rapid response). For 19 species, the low PPFD used was 100 μmol m^−2^ s^−1^, and high PPFD was 1000 μmol m^−2^ s^−1^. However, for four species (*S. plana*, *A. filix‐femina, O. regalis* and *A. thaliana*; denoted by ‘*’ in Table 1 and throughout) it was found that there was little increase in stomatal conductance rates at light levels above 100 μmol m^−2^ s^−1^, suggesting that stomata were already open. For these species, which we categorise as ‘low‐light adapted’, the low and high light levels used were 20 μmol m^−2^ s^−1^ and 500 μmol m^−2^ s^−1^. For *Z. mays* (which was grown at 1000 μmol m^−2^ s^−1^), a second set of measurements was taken to ensure that the full range of stomatal opening was achieved, in which low and high light were 100 and 2000 μmol m^−2^ s^−1^, respectively (denoted by ‘^’ and shown in Supporting Information).

### Stomatal dynamics calculations

To investigate the stomatal responses to step changes in PPFD, a previously described Excel macro for an asymmetric sigmoid response model was used (Vialet‐Chabrand *et al*., 2017) following the method outlined by McAusland *et al*. (2016).

(Eqn 1) $$g_{s}=\left(g_{\text{smax}}-r_{0}\right)e^{-e^{\left(\frac{\lambda -t}{k}+1\right)}}+r_{0}$$

This model comprised an initial *g*_s_ value at low PPFD and final *g*_s_ value at high PPFD (*r*_0_ and *g*_smax_, respectively), a time constant (*k*) denoting the time taken to achieve 63% of the change in *g*_s_ and a time lag (*λ*) to describe the time before *g*_s_ started to increase in response to the change in light flux. Using these parameters, the maximal rate of stomatal opening/closing responses was calculated as the maximum slope of the sigmoidal response (Sl_max_):

(Eqn 2) $${\mathrm{Sl}}_{\max}=\frac{k.\left(g_{\text{smax}}-r_{0}\right)}{e}$$

In the cases of species that ‘overshot’ during stomatal opening (seen by an initial increase in *g*_s_ under high light followed by a decrease to a constant value), the initial increase was modelled. Artificial data points within the *g*_s_ values caused by PPFD adjustment within the Li6800 were removed. Additionally, for one repeat of *G. biloba*, the curve fitting tool greatly overestimated maximum *g*_s_ during opening, and so the number of data points used was reduced to improve the fit.

### Growth under fluctuating light

Controlled environment growth chambers (Conviron PGR15; Conviron, Winnipeg, MB, Canada) delivered either 16 h of constant ‘day’ light at 400 μmol m^−2^ s^−1^ or fluctuating ‘day’ light, and 8 h night. The fluctuating light chamber was designed to mimic conditions recorded at a weather station at the Arthur Willis Environment Centre, University of Sheffield, S10 1AE, UK in June 2020. The readings from three highly light variable days were adjusted so that the daylength was 16 h and the daily light flux was equivalent to an average of 400 μmol m^−2^ s^−1^. Day : night temperature and humidity were set at 21°C : 16°C and 60%, respectively. *Triticum aestivum, T. durum, T. boeoticum, T. araraticum, B. distachyon, G. max* and *S. lycopersicum* seeds were sown into Levington's M3 potting compost (ICL, Suffolk, UK), and after 10 d seedlings were transplanted into pots (2 l for grasses and 6 l for *S. lycopersicum* and *G. max*) containing 6 : 1,M3 compost : perlite (Sinclair Pro, Cheshire, UK) and 5 g Osmocote Exact 5–6 slow‐release fertiliser (ICL, Suffolk, UK) per pot.

To calculate soil water capacity, additional pots containing the same mass of soil mixture were either dried in an oven for 5 d or saturated with water for 24 h before weighing. Five‐week‐old plants were weighed in their pots every day for 5 d. The water mass lost was recorded before re‐watering back to 70% field capacity. After 7 wk of growth in either constant or fluctuating light, aboveground biomass was dried for 2 wk at 60°C before weighing.

### Carbon isotope discrimination

Leaves from 7‐wk‐old plants (leaf 7 for grasses; 5^th^ node, middle leaf for *G. max* and *S. lycopersicum*), were dried for 2 wk and homogenised in a tissue lyser. About 1–2 mg was sealed in a tin capsule and placed into an isotope ratio mass spectrometer (IRMS, Sercon, UK) to determine the δ^13^C (carbon isotope composition) relative to the PeeDee belemnite carbonate standard (PDB). Eqn 3 was used to calculate Δ^13^C. Values for $\delta^{13}C_{\mathrm{air}}$ can be found in Table S2.

(Eqn 3) $$\Delta^{13}C=\frac{\delta^{13}C_{\mathrm{air}}-\delta^{13}C_{\text{plant}}}{1+\delta^{13}C_{\text{plant}}}$$

### Data analysis

R (v.3.5.3) was used for calculations and graph plotting. Significant differences across groups were identified using a Kruskal–Wallis test followed by a *post hoc* Dunn's test, as the data did not fit the assumptions for ANOVA. Comparisons between plants grown at fluctuating or constant light were carried out using an unpaired, two‐tailed *t*‐test. A Pearson correlation was used to determine the relationship between stomatal speed metrics and other parameters. A phylogenetic correction was applied to correlations using the phylogenetic generalised least squares (PGLS) method to perform the analysis using the caper package in R (Orme *et al*., 2025). By applying a maximum likelihood (ML) estimate to estimate the amount of phylogenetic correction required, it was found that the effect of phylogenetic signal on the correlations was negligible (see Table S3 for results of analysis). This is often the case for datasets containing < 30 species (Freckleton *et al*., 2002). Significance was assumed if *P* < 0.05.

## Results

### Stomatal morphologies across plant clades

Twenty‐three plant species were selected for the study, spanning several land plant evolutionary clades (Fig. 1; Table 1). Scanning electron microscopy showed a diversity in stomatal morphologies (Fig. S1); some species had stomata that protruded from the epidermal surface, whereas others were sunken (e.g. *A. filix‐femina* in comparison to *G. biloba*). As expected, lycophytes, ferns and some angiosperm species had kidney‐shaped guard cells. The shape of these stomata was variable and ranged from very rounded to elongated structures (e.g. *M. grandiflora* in comparison with *P. americana*). By contrast, species of the monocot grass clade had stomata with dumbbell‐shaped guard cells, which were less variable in morphology and consistently elongated in shape with distinct lateral subsidiary cells. The monocot species belonging to the Commelinales, *T. rubra*, had a somewhat intermediate stomatal morphology, having elongated kidney‐shaped guard cells plus subsidiary cells.

### Stomatal distributions across leaf surfaces

**Fig. 2:**
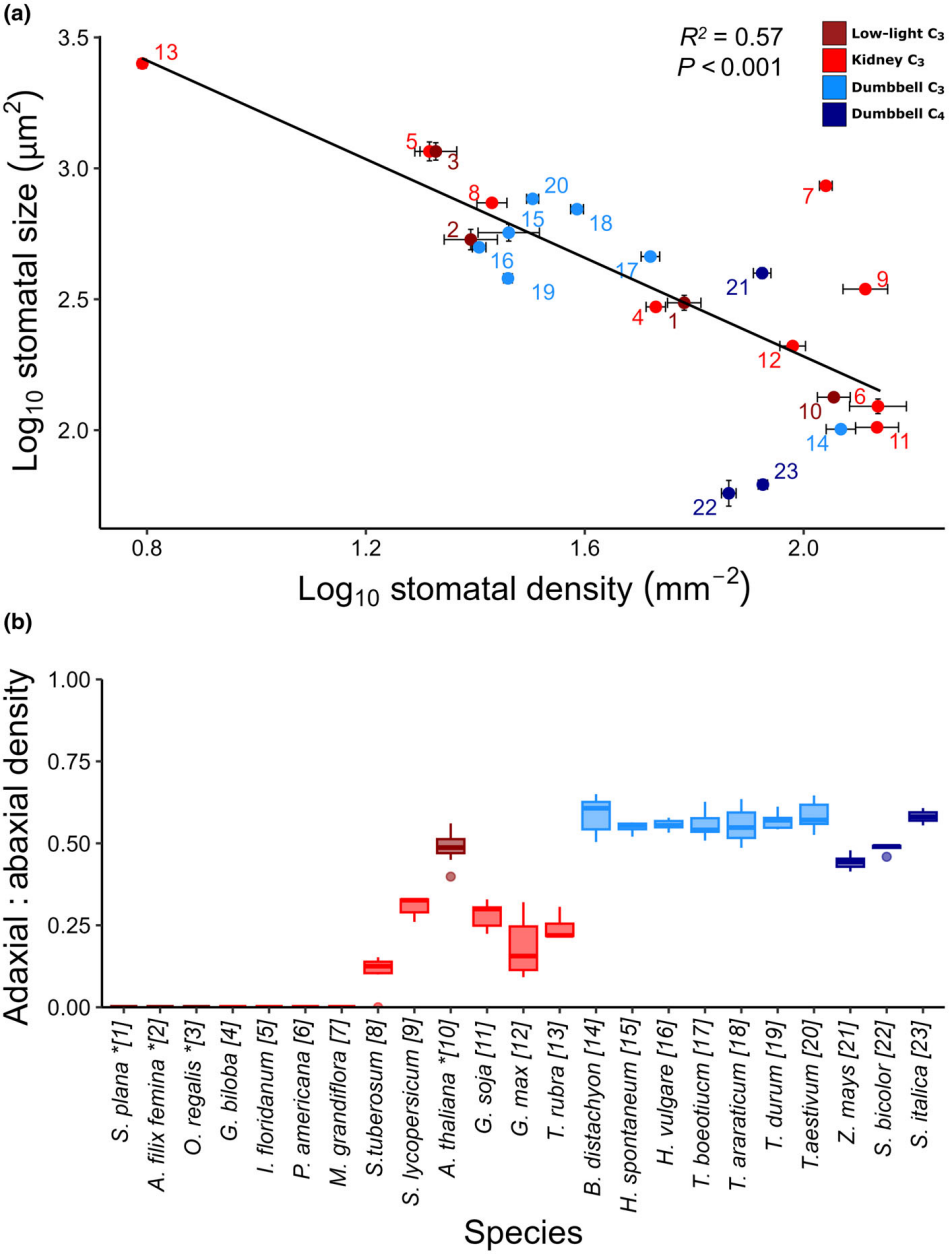
Stomatal size and distribution between leaf surfaces. (a) Correlation of mean stomatal complex areas and densities across species (both log_10_ tr). Mean density of stomata on abaxial and adaxial surfaces (where present) was plotted against mean size calculated from both abaxial and adaxial surfaces (Pearson correlation, *R* ^2^ = 0.57, *P* = 0.001). Error bars indicate SE. (b) Stomatal ratios (adaxial/abaxial density). *n* = 3–8 plants per species. Box plots indicate median and interquartile range. Box colours indicate species groups: dark red, kidney (low light) C_3_; light red, kidney (high light) C_3_; light blue, dumbbell C_3_; dark blue, dumbbell C_4_. Species ordered by phylogeny. Numbers indicated on (a) match species order on axis in (b) and Table 1 to allow species identification. Asterisks indicate low‐light species. *Selaginella plana*, *Athyrium filix‐femina*, *Osmunda regalis*, *Ginkgo biloba*, *Illicium floridanum*, *Persea americana*, *Magnolia grandiflora*, *Solanum tuberosum*, *Solanum lycopersicum*, *Arabidopsis thaliana*, *Glycine soja*, *Glycine max*, *Tradescantia rubra*, *Brachypodium distachyon*, *Hordeum spontaneum*, *Hordeum vulgare*, *Triticum boeoticu*, *Triticum araraticum*, *Triticum durum*, *Triticum aestivum*, *Zea mays*, *Sorghum bicolor*, *Setaria italica*.

Light microscopy revealed significant variation in stomatal size and density across the species (Fig. 2a,b). There was a *c*. 43‐fold difference between the largest and smallest mean stomatal complex areas measured: *T. rubra* (2522 μm^2^) and the C_4_ dumbbell‐shaped grass species *S. bicolor* (59 μm^2^). It should be noted that complex areas for *G. biloba* and *A. filix‐femina* may be slightly underestimated due to their sunken nature. Stomatal density varied 23‐fold across species, with the highest and lowest mean stomatal densities both measured from species with kidney‐shaped guard cells (*P. americana* at 142 mm^−2^ and *T. rubra* at 6.19 mm^−2^). In general, the species with dumbbell‐shaped guard cells were less diverse in their stomatal size and density than those with kidney‐shaped guard cells (Figs S1, S2). As observed previously (Grubb *et al*., 1975; Franks *et al*., 2009; Haworth *et al*., 2023), there was a significant negative correlation between stomatal size and density (Pearson correlation, *R* ^2^ = 0.57, *P* < 0.001 when log transformed; Fig. 2a). Amongst the grasses, the C_4_ species tended to have higher densities of small stomata in comparison with C_3_ grasses, as previously noted (Sage, 2004; Zhao *et al*., 2022; Zhou & Osborne, 2024).

Although there was no particular trend towards increased or decreased stomatal density nor size across evolutionary clades (Fig. S2), the distribution of stomata between the abaxial and adaxial surfaces of the leaf, known as the stomatal ratio, was strikingly grouped (Fig. 2b). Species belonging to the lycophytes, ferns, gymnosperms and angiosperms belonging to the ANA grade or Magnoliids (sister to the remaining angiosperms) all had stomata only on their abaxial leaf surfaces (i.e. hypostomatous), whereas the members of the later diverging angiosperms (*Asterids*, *Rosids*) and all monocots that we studied formed stomata on both leaf surfaces (amphistomatous). The *Asterid* and *Rosid* species were somewhat intermediate, with a higher proportion of stomata on the abaxial surface of the leaf. Grass species also had stomata on both surfaces but had slightly higher densities on the adaxial surface.

### Stomatal conductance and photosynthesis

**Fig. 3:**
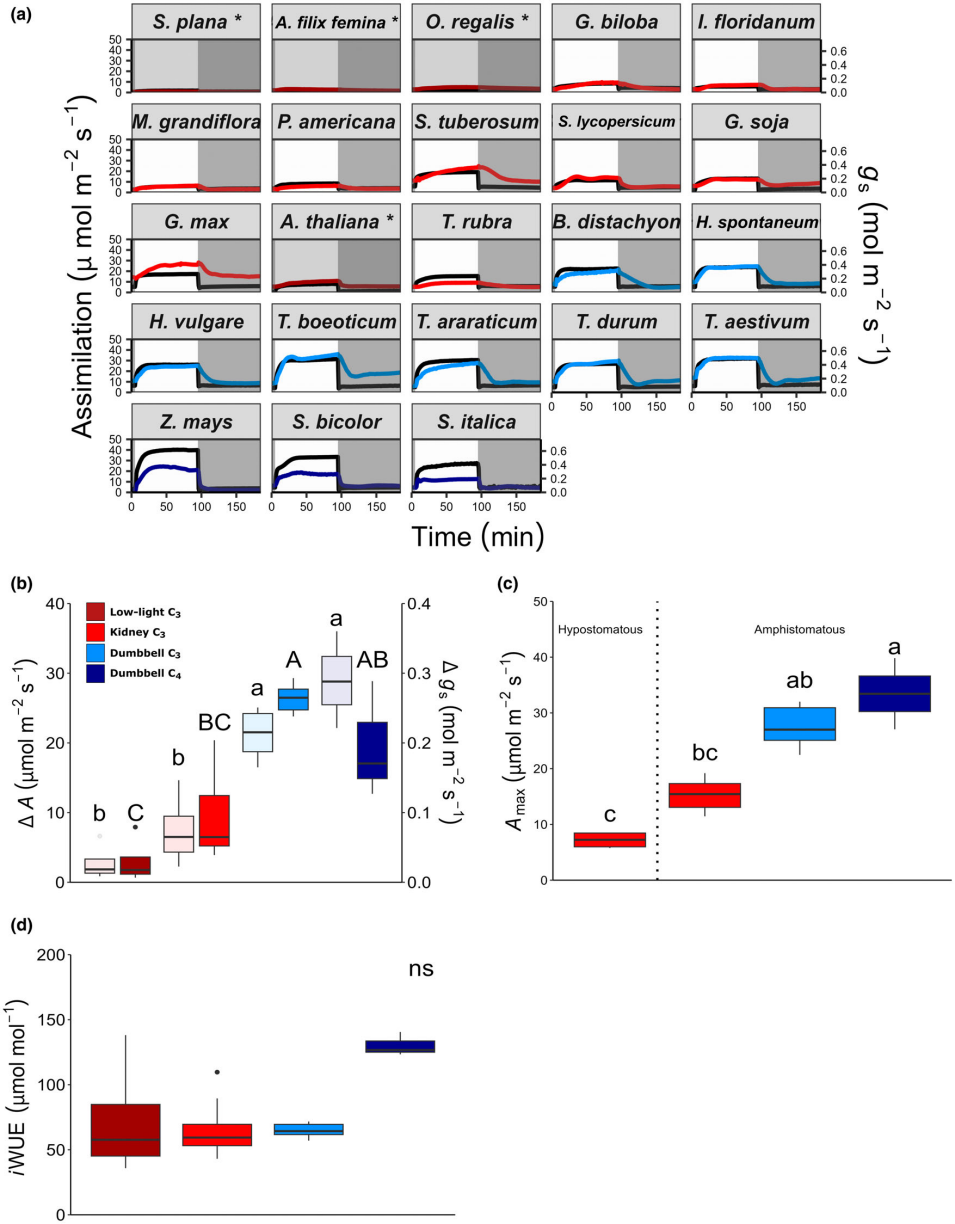
Stomatal conductance and photosynthesis rates. (a) Response of stomatal conductance (*g*_s_) and assimilation (*A*; grey) to a change in light intensity at 5 min from 100 μmol m^−2^ s^−1^ (shaded area) to 1000 μmol m^−2^ s^−1^ for 90 min, followed by a decrease to 100 μmol m^−2^ s^−1^ for 90 min (20 μmol m^−2^ s^−1^ to 500 μmol m^−2^ s^−1^ to 20 μmol m^−2^ s^−1^) for low light adapted species (*). (b) Change in *A* (light shaded boxes and lower‐case letters) and *g*_s_ (darker shaded boxes and uppercase letters) during stomatal opening. (c) Maximum recorded A at high light for species grouped by stomatal distribution, morphology and photosynthetic mechanism. (d) *i*WUE achieved during high light intensity. Box plots indicate median and interquartile range. Species that cannot be distinguished from each other at a 0.05 confidence limit are indicated by the same letters as determined by the Kruskal–Wallis test with a Dunn's test. ns, not significant across all groups. *n* = 3–8 per species. Line and box colours indicate species groups: dark red, kidney (low light) C_3_; light red, kidney (high light) C_3_; light blue, dumbbell C_3_; dark blue, dumbbell C_4_. *Selaginella plana*, *Athyrium filix‐femina*, *Osmunda regalis*, *Ginkgo biloba*, *Illicium floridanum*, *Persea americana*, *Magnolia grandiflora*, *Solanum tuberosum*, *Solanum lycopersicum*, *Arabidopsis thaliana*, *Glycine soja*, *Glycine max*, *Tradescantia rubra*, *Brachypodium distachyon*, *Hordeum spontaneum*, *Hordeum vulgare*, *Triticum boeoticu*, *Triticum araraticum*, *Triticum durum*, *Triticum aestivum*, *Zea mays*, *Sorghum bicolor*, *Setaria italica*.

Stomatal conductance and photosynthetic rates were measured for each species by IRGA at high and low PPFD and during the transitions between the two light flux levels (Fig. 3a). The lycophyte and fern species (all low‐light adapted species) had the lowest rates of *g*_s_ and *A*, whilst the grass species reached typically higher values, with the highest rates being achieved in the C_4_ cereal crop, *Z. mays* (both when measured at a PPFD of either 1000 or 2000 μmol m^−2^ s^−1^).

All species increased and decreased *g*_s_ and *A* in response to an increase or decrease in light flux to the adaxial surface, but there was a substantial difference in the magnitude of their responses (Fig. 3a). To investigate further, results were divided into species groups based on their guard cell morphology and photosynthetic mechanism. First, species were categorised as having either kidney or dumbbell‐shaped guard cells (shown in red and blue). Then, those with kidney‐shaped guard cells were subdivided into low‐light and high‐light adapted, and those with dumbbell‐shaped guard cells were subdivided into C_3_ or C_4_ photosynthetic type (see Tables 1, S1). These groups are referred to as ‘low‐light C_3_’, ‘kidney C_3_’, ‘dumbbell C_3_’ and ‘dumbbell C_4_’ in the figures and text below. The magnitude of both the *g*_s_ and *A* responses to light flux was significantly larger in the groups of species with dumbbell rather than in kidney‐shaped guard cells (Fig. 3b; for *A* (light‐shaded boxes), Kruskal–Wallis *P* < 0.001; for *g*_s_ (dark‐shaded boxes), Kruskal–Wallis, *P* < 0.001). There was a trend towards a decreased response in low‐light C_3_ species, and an increased photosynthetic response in dumbbell C_4_ species, although these were not statistically distinguishable.

The maximal levels of *g*_s_ and *A* at high light ranged from a mean of 0.073 and 3.77 μmol m^−2^ s^−1^, respectively, in low‐light adapted species, and up to 0.429 and 33.4 μmol m^−2^ s^−1^, respectively, in species with dumbbell‐shaped guard cells (i.e. *c*. 7‐fold and 13‐fold ranges in *g*_smax_ and *A*_max_). Further analysis, omitting the low‐light adapted species, revealed two noticeable uplifts in mean stomatal conductance and photosynthetic capacity (*A*_max_) between the earlier and later diverging angiosperm species. These step changes in *A*_max_ coincided with the acquisition of amphistomaty and the emergence of dumbbell‐shaped guard cells (Fig. 3c). Photosynthetic rates at 1000 μmol m^−2^ s^−1^ light were substantially higher in the angiosperm species with dumbbell‐shaped guard cells (in blue) than those with kidney‐shaped guard cells (in red), and there was a trend towards photosynthetic rate being higher still in the dumbbell C_4_ species (in dark blue; Kruskal–Wallis, *P* < 0.01).

### Water use efficiency

Calculation of intrinsic water use efficiency (*i*WUE) from gas exchange values at high PPFD revealed no differences in *i*WUE at steady state between species groups with kidney (C_3_) and dumbbell guard cells (C_3_ or C_4_), despite the latter group's significantly higher *g*_s_ and *A* values. The results were consistent with a rise in *i*WUE associated with the emergence of C_4_ photosynthesis and an accompanying decrease in *g*_s_; however, these differences were not statistically significant (Fig. 3d; Kruskal–Wallis, *P* > 0.05). *i*WUE measured during the first 30 min of stomatal opening or closure showed a similar trend with no significant difference between kidney or dumbbell C_3_ species, but dumbbell C_4_ species displayed a significantly higher *i*WUE during opening than low‐light‐adapted species (Fig. S3; Kruskal–Wallis, *P* < 0.05).

### Stomatal opening and closing responses

**Fig. 4:**
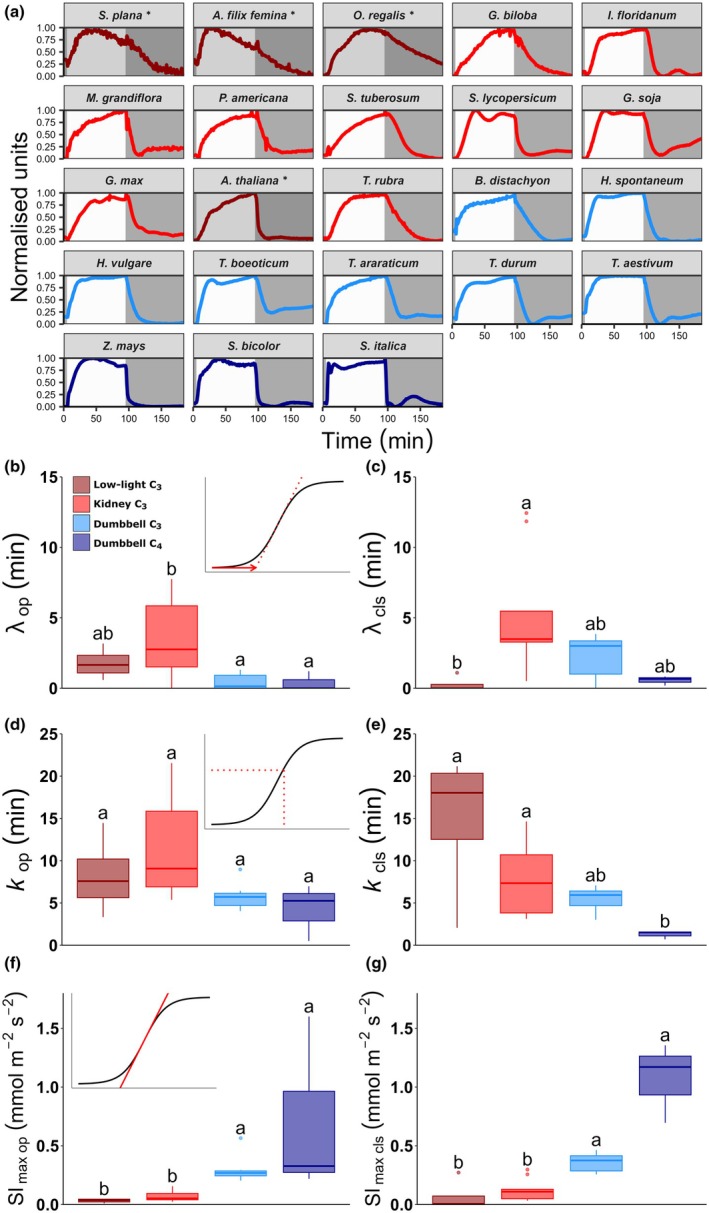
Stomatal response speeds. (a) Normalised stomatal conductance traces from Fig. 3(a) to allow comparison of the kinetics of responses. Speed parameters extracted from normalised data: (b, c) lag times before stomatal opening and closure (*λ*); (d, e) time to achieve 63% change in *g*_s_ during stomatal opening and closure (*k*); (f, g) maximal rate of change of *g*_s_ during stomatal opening and closure (Sl_max_). Box plots indicate median and interquartile range. Inset panels at top of (a, c, e) illustrate the nature of the *λ*_op_, *k*_op_ and Sl_max op_ parameters. Groups that cannot be distinguished from each other at a 0.05 confidence limit are indicated by different letters as determined by the Kruskal–Wallis test with a Dunn's test. *n* = minimum of three species per group. Line and box colours indicate species groups: dark red, kidney (low light) C_3_; light red, kidney (high light) C_3_; light blue, dumbbell C_3_; dark blue, dumbbell C_4_. *Selaginella plana*, *Athyrium filix‐femina*, *Osmunda regalis*, *Ginkgo biloba*, *Illicium floridanum*, *Persea americana*, *Magnolia grandiflora*, *Solanum tuberosum*, *Solanum lycopersicum*, *Arabidopsis thaliana*, *Glycine soja*, *Glycine max*, *Tradescantia rubra*, *Brachypodium distachyon*, *Hordeum spontaneum*, *Hordeum vulgare*, *Triticum boeoticu*, *Triticum araraticum*, *Triticum durum*, *Triticum aestivum*, *Zea mays*, *Sorghum bicolor*, *Setaria italica*.

For most species, photosynthesis increased more rapidly than stomatal conductance on light induction, but this was not always the case (Fig. 3a). For example, *S. plana* and *M. grandiflora* increased both *g*_s_ and *A* at similarly slow rates and several grass species, such as *T. aestivum*, increased both at similarly fast rates. In general, hypostomatous species had slower stomatal opening, and this appeared to limit the rise in photosynthesis in these species, whereas amphistomatous species showed a more rapid rise in *A* than *g*_s_. To aid comparison of the kinetics of stomatal responses to a change in PPFD, the stomatal conductance data were normalised to maximal values (Fig. 4a). Stomatal opening and closing speeds were each quantified in three ways, as shown in Fig. 4(b–g): (1) the lag times before an opening or closing response commenced (*λ*_op_ and *λ*_cls_); (2) the time taken to achieve 63% of maximal opening or closing after the initial lag phase (*k*_op_ and *k*_cls_); and (3) the maximum rates of change in conductance during opening or closing (Sl_max op_ and Sl_max cls_). These parameters were compared between species and plant groups (Figs 4, S4, S5). The lag time before stomatal opening, *λ*_op_, varied from *c*. 0 to 10 min across species (Kruskal–Wallis; *P* < 0.0001), and this was more variable amongst kidney C_3_ species (Fig. S5). All dumbbell C_3_ and C_4_ species were characterised by short *λ*_op_ lag times of < 2 min, and more than half of the grass species responded virtually instantaneously to light and did not have any measurable *λ*_op_. To facilitate further comparison, species were again divided into groups. Angiosperm dumbbell C_3_ and C_4_ species were found to have significantly shorter lag times before opening than angiosperm kidney C_3_ species (Kruskal–Wallis, *P* < 0.05; Fig. 4b) but there was no significant difference between *λ*_op_ of low‐light C_3_ and high‐light C_3_ species, nor between C_3_ and C_4_ dumbbell species. There was also variation in *λ*_cls_ across species (Kruskal–Wallis; *P* < 0.0001) with lag periods of between *c*. 0 and 13 min before starting to close, but *λ*_cls_ did not vary significantly between species when grouped by morphology or photosynthetic mechanism, with the exception of low‐light adapted species which had a shorter lag than high‐light C_3_ kidney species (Kruskal–Wallis; *P* < 0.05; Fig. 4c). The lycophyte and fern species all showed notably longer lags before commencing stomatal opening than closing, but this did not appear to be the case for *A. thaliana*, the other low‐light species.

The opening and closing time constants both varied significantly across species (Figs S4, S5, *k*_op_; Kruskal–Wallis; *P* < 0.001 and *k*_cls_; Kruskal–Wallis; *F*_(24,93)_ = 17.8, *P* < 0.001) with the slowest species (*G. biloba*) taking up to *c*. 22 min to reach 63% of opening or closing. Notably, the C_4_ grass, *S. italica*, was the fastest to reach 63% opening and closing, achieving both in less than a minute. However, *Z. mays* had slower kinetics and, on average, the C_4_ species group neither closed nor opened in a significantly shorter time than the C_3_ species. There was no obvious relationship between the speed of photosynthetic induction and the speed of stomatal opening or closing, and there was no clear correlation between opening and closing times; some species took a shorter time to open than close (e.g. the lycophyte and ferns and grasses), whereas others were faster to close than open (e.g. *A. thaliana*). Overall, species with dumbbell‐shaped guard cells did not take a significantly shorter time to achieve 63% opening (*k*_op_; Kruskal–Wallis, *P* > 0.05) nor closure (*k*_cls_; Kruskal–Wallis, *P* < 0.05; Fig. 4d,e) than those with kidney‐shaped guard cells, but dumbbell C_4_ species did have a significantly shorter *k*_cls_ than low‐light and C_3_ kidney species.

The maximum rates of stomatal opening and closing (Sl_max op_ and Sl_max cls_) varied significantly across species (Kruskal–Wallis; *P* < 0.001; Kruskal–Wallis; *P* < 0.001) (Figs S4, S5). When grouped by guard cell morphology or photosynthetic biochemistry, dumbbell C_4_ species were clearly able to close their stomata more rapidly than they opened them (except for *S. italica*, which was extremely fast at both opening and closing). Dumbbell C_4_ species also opened their stomata on average 9‐fold faster than species with kidney C_3_ species (mean values of 0.72 mmol m^−2^ s^−2^ for C_4_ vs 0.08 mmol m^−2^ s^−2^; Kruskal–Wallis, *P* < 0.001; Fig. 4f) but this was not statistically quicker than dumbbell C_3_ species. Dumbbell C_4_ species achieved the fastest Sl_max cls_, whilst low‐light C_3_ species achieved the slowest Sl_max cls_. On average, dumbbell C_4_ species achieved maximum stomatal closure rates that were 110‐fold faster than low‐light C_3_ species with kidney‐shaped guard cells (mean values of 1.1 mmol m^−2^ s^−2^ for C_4_ vs 0.01 mmol m^−2^ s^−2^; Kruskal–Wallis, *P* < 0.01; Fig. 4g).

The comparisons in Fig. 4(b,f) indicate that the acquisition of dumbbell‐shaped guard cells allowed grasses to begin to open their stomata almost immediately as light levels rise, and at a significantly faster maximal opening rate than angiosperm kidney C_3_ species. However, neither dumbbell C_3_ nor dumbbell C_4_ had a significantly shorter stomatal opening time constant *K*_op_ than kidney C_3_ (Fig. 4f). Conversely, when light levels decreased, C_3_ dumbbell morphologies did not have a significantly shorter lag time before closure commenced, nor did they have a shorter *K*_cls_, but they were able to achieve a faster maximum rate of stomatal closure, particularly in combination with C_4_ biochemistry. Our results did not reveal any obvious differences in any stomatal opening parameters associated with the acquisition of C_4_ photosynthesis. There was a trend towards C_4_ photosynthesis being associated with improvements in stomatal closure, but the mean values were not significantly different from C_3_ dumbbell species.

In addition to their dumbbell‐shaped guard cells, stomata of grass species also possess highly specialised subsidiary cells, which have been shown to be important for stomatal closure (Raissig *et al*., 2017). However, the presence of subsidiary cells in two nongrass species tested here (*G. biloba* and *T. rubra*) did not appear to enhance any of the stomatal response parameters in comparison with the other kidney C_3_ species (Fig. 4a).

### Relationship between stomatal size, distribution and response

**Fig. 5:**
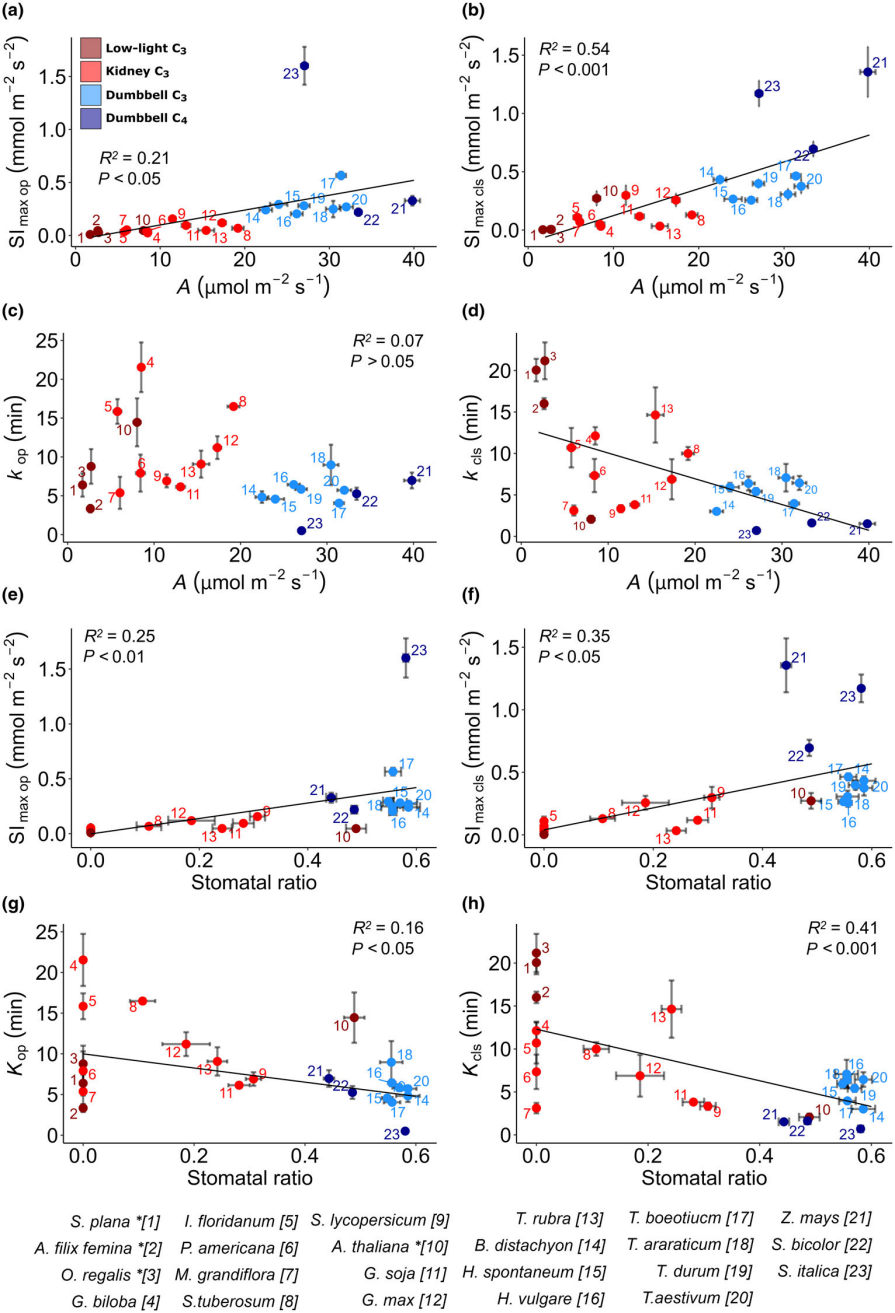
Relationship between stomatal speed, distribution and photosynthetic rate. Correlations between maximum *A* and stomatal opening and closing parameters: (a) Sl_max op_ (Pearson correlation, *R* ^2^ = 0.21, *P* < 0.05), (b) Sl_max cls_ (Pearson correlation, *R* ^2^ = 0.54, *P* < 0.001), (c) *k*_op_ (Pearson correlation, *R* ^2^ = 0.07, *P* < 0.05) and (d) *k*_cls_ (Pearson correlation, *R* ^2^ = 0.34, *p* < 0.01). Correlations between stomatal ratio and stomatal opening and closing parameters: (e) Sl_max op_ (Pearson correlation, *R* ^2^ = 0.25, *P* < 0.01); (f) Sl_max cls_ (Pearson correlation, *R* ^2^ = 0.35, *p* < 0.05); (g) *k*_op_ (Pearson correlation, *R* ^2^ = 0.16, *p* < 0.05); (h) *k*_cls_ (Pearson correlation, *R* ^2^ = 0.41, *P* < 0.001). Error bars indicate SE. Symbol colours indicate species groups: dark red, kidney (low light) C_3_; light red, kidney (high light) C_3_; light blue, dumbbell C_3_; dark blue, dumbbell C_4_. In each panel, numbered data points indicate species as shown in the figure and in Table 1. Asterisk indicates low‐light species. *Selaginella plana*, *Athyrium filix‐femina*, *Osmunda regalis*, *Ginkgo biloba*, *Illicium floridanum*, *Persea americana*, *Magnolia grandiflora*, *Solanum tuberosum*, *Solanum lycopersicum*, *Arabidopsis thaliana*, *Glycine soja*, *Glycine max*, *Tradescantia rubra*, *Brachypodium distachyon*, *Hordeum spontaneum*, *Hordeum vulgare*, *Triticum boeoticu*, *Triticum araraticum*, *Triticum durum*, *Triticum aestivum*, *Zea mays*, *Sorghum bicolor*, *Setaria italica*.

To test the hypothesis that stomatal size is an important contributor to stomatal response speed, a covariance analysis between stomatal patterning characteristics (Fig. 2b) and gas exchange response parameters (Figs 3, 4) was conducted. Perhaps surprisingly, no significant correlations were found between either *k* or Sl_max_ values and stomatal size across all species during either opening or closing, except for *k*_cls_, which was weakly positively correlated with stomatal size (Fig. S6; Pearson correlation, *R* ^2^ = 0.21, *P* < 0.05). By contrast, we observed several strong relationships between stomatal speed parameters and photosynthetic rate (Fig. 5). During both opening and closure, Sl_max_ was positively correlated with maximum *A* achieved under high PPFD. Whilst *K*_op_ and *A*_max_ showed no relationship (Fig. 5c), *A*_max_ was negatively correlated with *K*_cls_ (Fig. 5d). This relationship is highlighted by the fast maximal closure rates and short *K*_cls_ achieved by plants with C_4_ photosynthesis (dark blue in Fig. 5b,d). In contrast to *A*_max_, stomatal speed parameters did not correlate as strongly with *g*_smax_, presumably because of the lower *g*_s_ values in C_4_ plants, as can be seen in Fig. 3(b). Only *K*_cls_ and *g*_smax_ were significantly correlated (Pearson correlation, *R* ^2^ = 0.14, *P* < 0.05). It is worth noting that when a phylogenetic correction was applied to these correlations, the relationship between these stomatal response speed parameters and *A*_max_ was not significant (see Table S3 for details of significance values).

In contrast to stomatal size, we found significant correlations between stomatal distribution and stomatal speed, particularly in the closing response. Both Sl_max_ values for opening (Fig. 5e) and closing (Fig. 5f) were found to be positively correlated with stomatal ratio (Sl_max op_: Pearson correlation, *R* ^2^ = 0.25, *P* < 0.01; Sl_max cls_: Pearson correlation, *R* ^2^ = 0.35, *P* < 0.05). *K*_op_ (Pearson correlation, *R* ^2^ = 0.16, *P* < 0.05) and *K*_cls_ (Pearson correlation, *R* ^2^ = 0.41, *P* < 0.001) were negatively correlated with stomatal ratio (Fig. 5g,h).

### Role of stomatal response rates in a fluctuating light environment

**Fig. 6:**
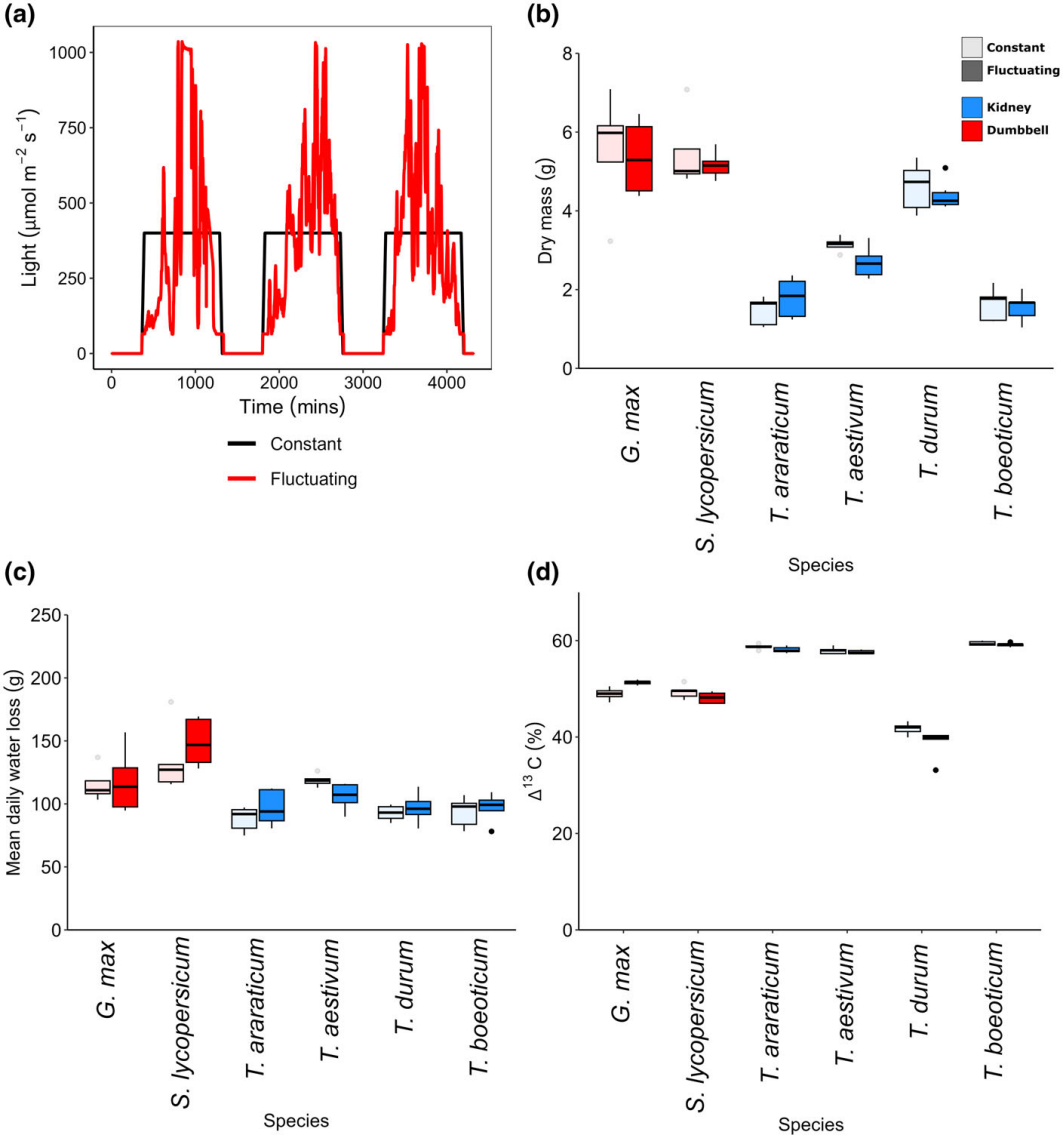
Growth and water use under constant or fluctuating light. (a) Diurnal light flux regime shown over 3 d in the constant (black) and fluctuating (red) growth chambers. (b) Dry mass after 7 wk of growth. (c) Daily water loss over 5 d. (d) Carbon isotope discrimination between plants grown under constant and fluctuating light. Box plots indicate median and interquartile range. Data shown are the mean ± SE. *n* = minimum of five plants per species. Symbol shading indicates light regime: Light, constant light; Dark, fluctuating light. Symbol colours indicate species groups: red, kidney; blue, dumbbell. Asterisk indicates significant difference at 0.05 confidence limit (unpaired *t*‐test). *Selaginella plana*, *Athyrium filix‐femina*, *Osmunda regalis*, *Ginkgo biloba*, *Illicium floridanum*, *Persea americana*, *Magnolia grandiflora*, *Solanum tuberosum*, *Solanum lycopersicum*, *Arabidopsis thaliana*, *Glycine soja*, *Glycine max*, *Tradescantia rubra*, *Brachypodium distachyon*, *Hordeum spontaneum*, *Hordeum vulgare*, *Triticum boeoticu*, *Triticum araraticum*, *Triticum durum*, *Triticum aestivum*, *Zea mays*, *Sorghum bicolor*, *Setaria italica*.

To understand whether stomatal response speeds can affect growth parameters in a fluctuating light environment, six species (with a *c*. 3‐fold range of *k*_op_ and *k*_cls_) were grown under either constant or fluctuating day‐time light conditions. *Solanum lycopersicum*, *G. max*, *T. boeoticum, T. araraticum*, *T. durum* and *T. aestivum* were chosen because they all have a C_3_ photosynthetic mechanism and grow well under the same conditions, but as described above, they had a range of differing guard cell morphologies and stomatal opening and closing speeds. To probe the importance of stomatal response speeds for water relations, these species were propagated under two light regimes, each with equal day‐lengths and total amounts of light delivered: one with a constant level of daylight and the other designed to mimic real‐life data (Fig. 6a). After 7 wk of growth, there was no difference in stomatal density between the two growth conditions for any species (unpaired *t*‐test, *P* > 0.05). We also observed no significant differences in plant dry biomass gained nor water lost for any individual species following growth under constant light in comparison with fluctuating light flux (unpaired *t*‐test, *P* > 0.05; Fig. 6b,c). Leaf carbon isotope discrimination (Δ^13^C) analysis was used as a proxy value to allow comparison of WUE across the lifetime of the tissue (Farquhar *et al*., 1989). There were no significant differences in leaf carbon isotope discrimination between constant and dynamic light regimes (unpaired *t*‐test, *P* > 0.05; Fig. 6d), for all species with the exception of *G. max*. This species, which had relatively long stomatal opening and closing times, had higher Δ^13^C under the fluctuating light environment, indicating a lower WUE (unpaired *t*‐test, *t* = 3.100, *P* < 0.01). These Δ^13^C measurements of WUE are in line with the finding that *i*WUE was not enhanced by the acquisition of dumbbell‐shaped guard cells, as shown in Fig. 3(d).

## Discussion

### Amphistomaty and dumbbell‐shaped guard cells support high levels of gas exchange and enable faster stomatal responses

We measured variation in the maximum levels and rates of change of *g*_s_ and *A* across species spanning several branches of the land plant phylogeny and found, as might be expected, that later diverging groups generally had much higher maximal rates of gas exchange (Fig. 3a). In most species, *A* increased and decreased at a much faster rate than *g*_s_, consistent with previous studies (Drake *et al*., 2013; McAusland *et al*., 2016; Deans *et al*., 2019; Wall *et al*., 2022). Our experiments included 10 crop species, three of which were paired with wild progenitors (Table 1). From the species studied, our results showed no trend towards either faster stomatal responses or higher photosynthetic rates in the domesticated species when compared with their progenitors. Instead, our data provide evidence for several step changes in stomatal *g*_s_ and *A* during evolution (Fig. 3c).

### Amphistomaty allows higher levels of gas exchange

The first major increase in *g*_s_ and *A* is associated with a change in plant development, producing stomata on both abaxial and adaxial leaf surfaces. It is known that both abaxial and adaxial stomata can contribute to photosynthesis, and that in wheat, the adaxial surface supports higher levels of *g*_s_ and *A* (Wall *et al*., 2022). In our experiments, amphistomaty was associated with a mean increase in *A* of *c*. 1.5‐fold in C_3_ kidney species and stomatal ratio was found to be correlated with stomatal speed parameters. Our data from a limited number of species suggest that earlier diverging clades, such as lycophytes and ferns, were predominantly hypostomatous, and amphistomaty appeared after the early angiosperms *c*. 150 Ma, in line with previous observations, although this conclusion is complicated by amphistomatous fossils of older extinct lineages (Bomfleur & Kerp, 2010), the occurrence of adaxial stomata on microphylls of some Selaginella species (Soni *et al*., 2012), and reversions to hypostomaty in some low‐light adapted species (Mott *et al*., 1982). Furthermore, our data indicate that angiosperm clades have arisen with a steadily increasing proportion of stomata on their adaxial leaf surfaces. The evolutionary appearance of amphistomaty is known to have been accompanied by other traits including an increase in vein density and thicker leaves facilitated by shorter pathway distances for CO_2_ to access the photosynthetic machinery, all features which would also be expected to enhance *g*_s_ and *A* (Feild & Brodribb, 2013; Drake *et al*., 2019). However, not all angiosperm species are amphistomatous (de Boer *et al*., 2016) and it would be interesting to also include hypostomatous angiosperm species in future studies.

### Dumbbell‐shaped guard cells support high levels of gas exchange and faster stomatal responses

In our experiments, the presence of dumbbell‐shaped guard cell morphology correlated with a second major increase in leaf gas exchange. This innovation in guard cell geometry occurred over 100 Ma and appears to support a further 2‐fold increase in rates of photosynthesis. A third increase in *A* (of *c*. 0.2‐fold and not significant in our experiments) is associated with the acquisition of C_4_ biochemistry, which has arisen multiple times over the past 20–30 Ma in the grasses (Sage, 2004). In addition to higher levels of *g*_s_ and *A*, dumbbell C_3_ and C_4_ species had higher maximal rates of both stomatal opening and closing than kidney C_3_ species, supporting the theoretical and empirical evidence regarding the advantages of these specialised dumbbell‐shaped cells (Hetherington & Woodward, 2003; Vico *et al*., 2011; McAusland *et al*., 2016).

Dumbbell‐shaped guard cells require a smaller change in turgor or width to adjust their relatively longer and more rectangular‐shaped pores (Hetherington & Woodward, 2003; Franks & Farquhar, 2007; Raven, 2014; Durney *et al*., 2023). Thus, in a given time, species with dumbbell‐shaped guard cells should achieve a greater change in *g*_s_ than kidney‐shaped guard cells. The time constant measuring the rapidity of the stomatal response, *k*, is independent of the amplitude of change (Vialet‐Chabrand *et al*., 2017) and provides a way to compare the time taken to adjust pore apertures. Many species with kidney C_3_ species achieved *k* values that were similar to dumbbell C_3_ species, perhaps indicating that the superior Sl_max_ values in dumbbell C_3_ (and C_4_) species are necessary because of the greater magnitude in the change of *g*_s_ achieved in these species. Thus, some of the success of species with dumbbell‐shaped guard cells may be attributable to other factors, such as the higher gas exchange levels supported by the more rectangular pore, rather than the ability to open and close in a short time period.

The C_4_ dumbbell species tested here showed generally higher Sl_max_ rates and shorter *k* and λ times of stomatal adjustment than C_3_ dumbbell species, in line with other studies (McAusland *et al*., 2016; Ozeki *et al*., 2022; Rui *et al*., 2024), although in our data, neither *k* value was significantly shorter. The evolution of C_4_ photosynthesis is thought to be an adaptation to conditions that favour photorespiration, such as drought (Sage, 2004; Bräutigam & Gowik, 2016), and the rapid stomatal closure response is likely an adaptation to reduce unnecessary water loss upon a PPFD reduction, whilst the carbon concentrating mechanism limits losses in *A*. The mechanism(s) by which C_4_ plants achieve faster stomatal responses remain unknown, but it has been suggested to be due to a smaller stomatal complex size and greater guard cell surface to volume ratio (Lawson & Blatt, 2014; Ozeki *et al*., 2022; Rui *et al*., 2024). No relationship was found here between stomatal complex size and Sl_max op_ or *k*_op_, although dumbbell C_4_ species did have relatively high values of Sl_max cls_. We therefore propose that the tight relationship that we identified here between Sl_max_ and *A*_max_ indicates that faster stomatal speeds in C_4_ species may be linked to their ability to support higher photosynthetic rates.

We also measured the time lag before a stomatal response (Fig. 4b,c). While dumbbell C_3_ and C_4_ species had lower λ_op_ values, this difference was not significant for λ_cls_, indicating that dumbbell‐shaped guard cells can trigger stomatal opening, but perhaps not closing, faster than kidney‐shaped guard cells. The reduced λ associated with dumbbell‐shaped guard cells has been previously noted, but the asymmetry between opening and closure has rarely been considered (McAusland *et al*., 2016; Wall *et al*., 2022).

### Stomatal size does not correlate with response speed across a broad range of species

It is widely accepted that smaller stomata should be able to open and close more quickly than larger stomata, with some experimental evidence supporting this (Drake *et al*., 2013; Kardiman & Ræbild, 2018; Israel *et al*., 2022; Ozeki *et al*., 2022; Zhang *et al*., 2022). However, studies comparing distantly related species or those with distinctly different ecological niches do not show a strong correlation, if any, between stomatal size and speed (Elliott‐Kingston *et al*., 2016; McAusland *et al*., 2016; Deans *et al*., 2019). Although our data show a clear correlation between the dumbbell‐shaped stomatal morphology and maximal stomatal opening and closure speeds (Fig. 4g), we found no relationship between either Sl_max_ or *k* values and stomatal size during either opening or closure, with the exception of a weak correlation with *k*_cls_. These data suggest that other guard cell features are important for determining response times. Differences in the levels of light‐sensing receptors such as phototropins and cryptochromes, or other signalling components, can lead to differences in stomatal response speeds (Cai *et al*., 2021), and differences in guard cell wall composition or stiffness can affect stomatal range and speed of adjustment (Jones *et al*., 2005; Amsbury *et al*., 2016; Shtein *et al*., 2017; Carroll *et al*., 2022). Thus, our results indicate that guard cell morphology is a more important contributor to stomatal speed parameters than stomatal size, but the relative contributions of signalling pathways and guard cell wall properties could also be important. However, stomatal size could exert a greater influence on stomatal kinetics in closely related species where guard cell morphology and other features might be expected to be more similar.

### Many species show an asymmetry between stomatal opening and closing responses

While some species consistently opened and closed their stomata more quickly than others, several showed a clear asymmetry between opening and closing responses. In particular, the ferns and lycophytes (*S. plana*, *A. filix‐femina* and *O. regalis*) opened their stomata in a shorter time than they closed them, consistent with a previous study on ferns (Deans *et al*., 2019). This appears to be an adaptation to light‐limited conditions of the understory environment where the ability to maximise photosynthesis during light flecks, by opening stomata in a short timeframe, is important (Knapp & Smith, 1987; Endler, 1993; Deans *et al*., 2019). In Polypodiale ferns (which include *A. filix‐femina*), duplications of cryptochrome genes are thought to enhance the stomatal response to blue light (Cai *et al*., 2021). In contrast to these understory species, both *Z. mays and S. bicolor* dumbbell C_4_ species achieved much higher Sl_max_ and shorter *k* values during closure than opening. This asymmetric response may be an adaptation to water‐limiting conditions with plentiful light, providing advantageous traits desirable in crop plants. However, this was not the case in *S. tuberosum* or *G. max* C_3_ kidney crop species.

### Faster stomata do not always result in a physiological advantage

It has been suggested that faster stomatal responses convey an advantage in a dynamic light environment by maximising *A* on sudden increases in light and minimising water loss during the onset of low light (Ooba & Takahashi, 2003; Vialet‐Chabrand *et al*., 2017). However, for five out of six species measured, we found no reduction in biomass acquisition, water loss nor WUE (estimated using carbon isotope discrimination) when plants were grown under fluctuating rather than constant light. Only *G. max*, which had the longest *k*_op_ in this experiment, showed a reduction in time‐integrated WUE (but no significant difference in biomass or water use). This contrasts with studies using increased stomatal speed mutants, which have found improvements in biomass and WUE efficiency. This may, in part, be due to the research being carried out in *A. thaliana*, which has a long *k*_op_ (the longest *k*_op_ amongst angiosperms in our study), or to the more simple alternating light regimes used in previous studies (Papanatsiou *et al*., 2019; Kimura *et al*., 2020). Our results indicate that for the majority of angiosperms, and in particular those with dumbbell‐shaped guard cells, more rapid stomatal responses do not confer any advantages in fluctuating light environments.

In line with previous studies, our work indicates that early diverging plants such as lycophytes and ferns often have stomata on their abaxial leaf surfaces only. Adaxial stomata appear in the early angiosperms and support higher levels of stomatal conductance and photosynthesis (except in species adapted to low‐light environments). Dumbbell‐shaped guard cells, which arose in the grasses, facilitate higher stomatal conductance and photosynthesis. They also improve some stomatal opening and closing parameters, shortening the lag period before stomata commence opening (*λ*_op_) and facilitating faster maximal rates of opening and closing (Sl_max_). The addition of C_4_ biochemistry allows even faster stomatal closure rates (Sl_max cls_).

Our results do not support the hypothesis that smaller stomata generally adjust their apertures within a shorter time nor at a faster rate. Instead, stomatal response speeds are more closely related to photosynthetic rates. In contrast to several previous studies, we did not find a correlation between smaller stomata and faster rates of either opening or closing, and we suggest that the geometry of the stomatal complex (i.e. dumbbell‐shaped guard cells), and in some cases the photosynthetic biochemistry, are more important than stomatal size in facilitating fast stomatal movements. By contrast, we identified a strong correlation between increasing photosynthetic rate and stomatal speed, which was associated with two evolutionary innovations in stomatal development: amphistomaty and dumbbell‐shaped guard cells. The significance of these correlations was not maintained when a phylogenetic correction was performed on the data, probably because of the small number of species used in this study. While we sampled species from across the land plant phylogeny, several crop and progenitor pairings were included, and grasses made up almost half of the species studied. Thus, it is unsurprising that the limited number and uneven phylogenetic distribution of species restricted our ability to make phylogenetically independent conclusions (Freckleton *et al*., 2002).

Perhaps surprisingly, we could not find any biological advantage to having faster stomatal opening or closure speeds. Species with faster stomatal adjustment did not show reduced water loss, improved WUE or greater biomass accumulation in a fluctuating light environment. This raises the question of why some plant species invest energy resources in speedier stomata. Further research is required to determine whether faster stomata can improve growth or survivability under field conditions, and it would be interesting to explore whether the faster stomatal speeds often associated with C_4_ plants can improve performance in fluctuating environments.

Although our results suggest that particularly slow stomatal opening may reduce WUE in some situations, they do raise the question of whether faster stomata arose for another reason. We propose that evolutionary innovations, including amphistomaty and dumbbell‐shaped guard cells, have primarily been selected during evolution because they facilitated step changes in the rate of stomatal conductance (and thereby photosynthesis) rather than primarily because of their ability to adjust more quickly to their environment. Thus, we conclude that the evolution of stomatal and biochemical adaptations that support higher levels of photosynthesis also facilitates improvements in stomatal speed, perhaps through the provision of the additional energy required to open stomata more frequently. This proposal is in line with findings that plants with higher levels of guard cell H^+^‐ATPase have increased stomatal opening and enhanced levels of photosynthesis and biomass (Wang *et al*., 2014) and that stomatal opening speed correlates with photosynthetic capacity in C_4_ plants (Zhou & Osborne, 2024).

## Competing interests

None declared.

## Author contributions

RAB, JEG and AJF planned and designed the research. RAB and MJW performed experiments. RAB, MJW and SJT analysed data. RAB, MJB, SJT, AJF and JEG wrote the manuscript.

## Disclaimer

The New Phytologist Foundation remains neutral with regard to jurisdictional claims in maps and in any institutional affiliations.

## Supporting information


**Fig. S1** Images of stomatal morphologies.
**Fig. S2** Stomatal sizes and densities for individual species.
**Fig. S3** *i*WUE during opening and closure.
**Fig. S4** Stomatal opening speed parameters for individual species.
**Fig. S5** Stomatal closing speed parameters for individual species.
**Fig. S6** Lack of relationship between stomatal size and speed parameters.
**Table S1** Plant growth and gas exchange conditions.
**Table S2** ^13^C_air_ values from fluctuating light experiment.
**Table S3** Regression statistics after phylogenetic correction.
Please note: Wiley is not responsible for the content or functionality of any Supporting Information supplied by the authors. Any queries (other than missing material) should be directed to the *New Phytologist* Central Office.

## Data Availability

The data that support the findings of this study are available in the Supporting Information of this article. Scripts used for data analysis are publicly available at https://doi.org/10.15131/shef.data.30608657.
