# Supplementary material for: Was the evolution of faster stomata driven by increased gas exchange rates rather than increasing water use efficiency?
Source: New Phytol. 2025 Dec 24;249(5):2355–71. doi: 10.1111/nph.70830 (PMC12873517; doi:10.1111/nph.70830)
Supplement: Supplementary file 1 — Fig. S1 Images of stomatal morphologies. Fig. S2 Stomatal sizes and densities for individual species. Fig. S3 iWUE during opening and closure. Fig. S4 Stomatal opening speed parameters for individual species. Fig. S5 Stomatal closing speed parameters for individual species. Fig. S6 Lack of relationship between stomatal size and speed parameters. Table S1 Plant growth and gas exchange conditions. Table S2 13Cair values from fluctuating light experiment. Table S3 Regression statistics after phylogenetic correction. Please note: Wiley is not responsible for the content or functionality of any Supporting Information supplied by the authors. Any queries (other than missing material) should be directed to the New Phytologist Central Office. [file NPH-249-2355-s001.pdf]

New Phytologist

***New Phytologist* Supporting Information**

Article title: Was the evolution of faster stomata driven by higher gas exchange rates rather than increasing water use efficiency?

Authors: Robert A. Brench, Matthew J. Wilson, Sarah J. Thorne, Andrew J. Fleming, Julie E. Gray

Article acceptance date: 20 November 2025

The following Supporting Information is available for this article:

Table S1. Plant growth and gas exchange conditions.

Table S2. $\delta^{13}C_{air}$ values from fluctuating light experiment.

Table S3. Regression statistics after phylogenetic correction

Figure S1. Images of stomatal morphologies.

Figure S2. Stomatal sizes and densities for individual species.

Figure S3. iWUE during opening and closure.

Figure S4. Stomatal opening speed parameters for individual species.

Figure S5. Stomatal closing speed parameters for individual species.

Figure S6. Lack of relationship between stomatal size and speed parameters.

New Phytologist

Table S1. Plant growth and gas exchange conditions. * Indicates species grown at low light (low-light adapted) and ^ indicates grown at light equal to maximum value during light shift.

Species	Growth conditions	Growth Medium	IRGA experimental material
*Selaginella plana**	10h light/ 14h dark, 100 µmol $m^{-2}s^{-1}$ PPFD, 22°C temperature.	Levington F2S and sand compost	Youngest expanded tissue.
*Athyrium filix-femina**<br>*Osmunda regalis**		Burncoose potting medium	Youngest fully expanded leaves of new growth in stated conditions for at least 1 month.
Illicium floridanum			
Magnolia grandiflora<br>*Ginkgo biloba*	14h light/ 10h dark, 600 µmol $m^{-2}s^{-1}$ PPFD, 22°C temperature.		Youngest fully expanded leaves of new growth in stated conditions for at least 1 month.
Solanum tuberosum	12h light/ 12h dark, 600 µmol $m^{-2}s^{-1}$ PPFD	6:1 M3: perlite	Middle leaf of node 5 of 5 week old plants.
Persea americana	12h light/ 12h dark, 600 µmol $m^{-2}s^{-1}$ PPFD, 30°C/ 25°C temperature.		Youngest fully expanded leaves.
Solanum lycopersicum<br>*Glycine max*	16h light/ 8h dark, 600 µmol $m^{-2}s^{-1}$ PPFD, 25°C/ 21°C temperature.		Middle leaf of node 5 of 5 week old plants.
*Arabidopsis thaliana**	12h light/ 12h dark, 125 µmol $m^{-2}s^{-1}$ PPFD, 22°C.		Youngest fully expanded leaves.
Glycine soja	16h light/ 8h dark, 400 µmol $m^{-2}s^{-1}$ PPFD, 21°C/ 16°C temperature.		
Tradescantia rubra	16h light/ 8h dark, 150 µmol $m^{-2}s^{-1}$ PPFD, 22°C temperature.		
Zea mays (^)	16h light/ 8h dark, 1000 µmol $m^{-2}s^{-1}$ PPFD, 28°C/ 20°C temperature.	5:1 John Innes No.3: Course horticultural sand	Leaf 7, avoiding the midrib.
Brachypodium distachyon<br>*Hordeum spontaneum*<br>*Hordeum vulgare*<br>*Triticum* species x 4<br>*Sorghum bicolor*<br>*Setaria italica*	16h light/ 8h dark, 400 µmol $m^{-2}s^{-1}$ PPFD, 21°C/ 16°C temperature.	6:1 M3: perlite	Middle of leaf 5 of 4-5 week old plants.

Table S2. $\delta^{13}C_{air}$ values from fluctuating light experiment. $\delta^{13}C_{air}$ values taken from inside the chamber of species grown under constant or fluctuating daylight conditions.

**Species**	**Chamber**	$\delta^{13}C_{air}$
Triticum aestivum	Constant daytime light	-13.84
Triticum araraticum	Constant daytime light	-13.84
Triticum boeotiucm	Constant daytime light	-13.84
Solanum lycopersicum	Constant daytime light	-15.28
Glycine max	Constant daytime light	-15.28
Triticum durum	Constant daytime light	-18.77
Triticum aestivum	Fluctuating daytime light	-13.68
Triticum araraticum	Fluctuating daytime light	-13.68
Triticum boeotiucm	Fluctuating daytime light	-13.68
Solanum lycopersicum	Fluctuating daytime light	-18.77
Glycine max	Fluctuating daytime light	-18.77
Triticum durum	Fluctuating daytime light	-19.11

Table S3. Regression statistics after phylogenetic correction. Regression statistics from A_{max} against stomatal opening and closing parameters after a phylogenetic correction was applied that assumed a Brownian motion model of evolution.

**Factor**	**Factor**	**R^2**	***p***
A_{max}	$Sl_{max\ op}$	0.19	0.39
A_{max}	TC_{op}	0.36	0.10
A_{max}	$Sl_{max\ cls}$	0.35	0.27
A_{max}	TC_{cls}	0.36	0.10

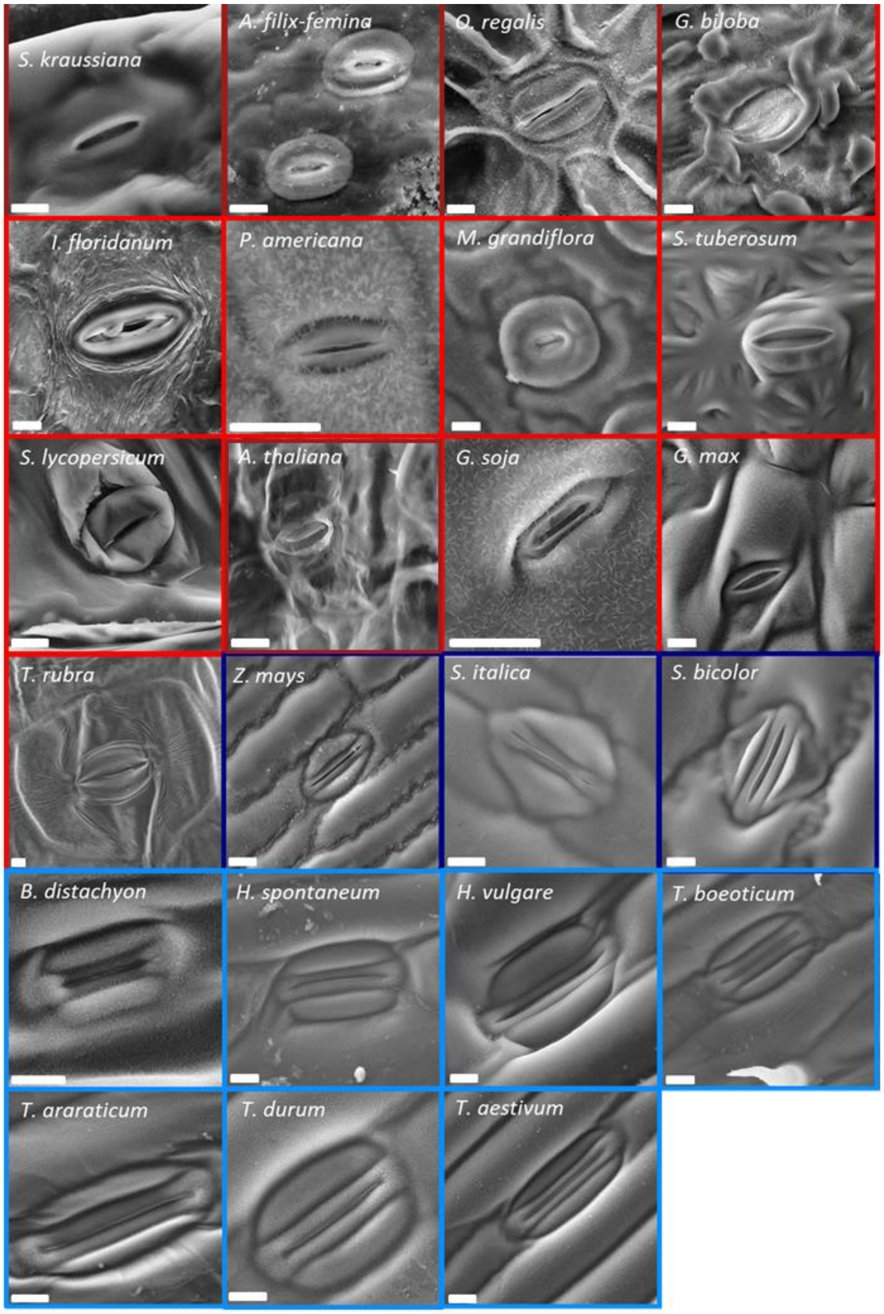

Fig. S1. Images of stomatal morphologies. Images were obtained with an environmental scanning electron microscope (SEM; Hitachi, Tokyo, Japan). Samples were mounted on adhesive stubs and imaged under a vacuum using the TM30303Plus application. *Selaginella krausianna* is shown instead of *Selaginella plana*. Scale bar = 10 µm. Border colours indicate species group: Kidney (low light) C3 = dark red; Kidney (high light) C3 = light red; Dumbbell C3 = light blue; Dumbbell C4 =dark blue.

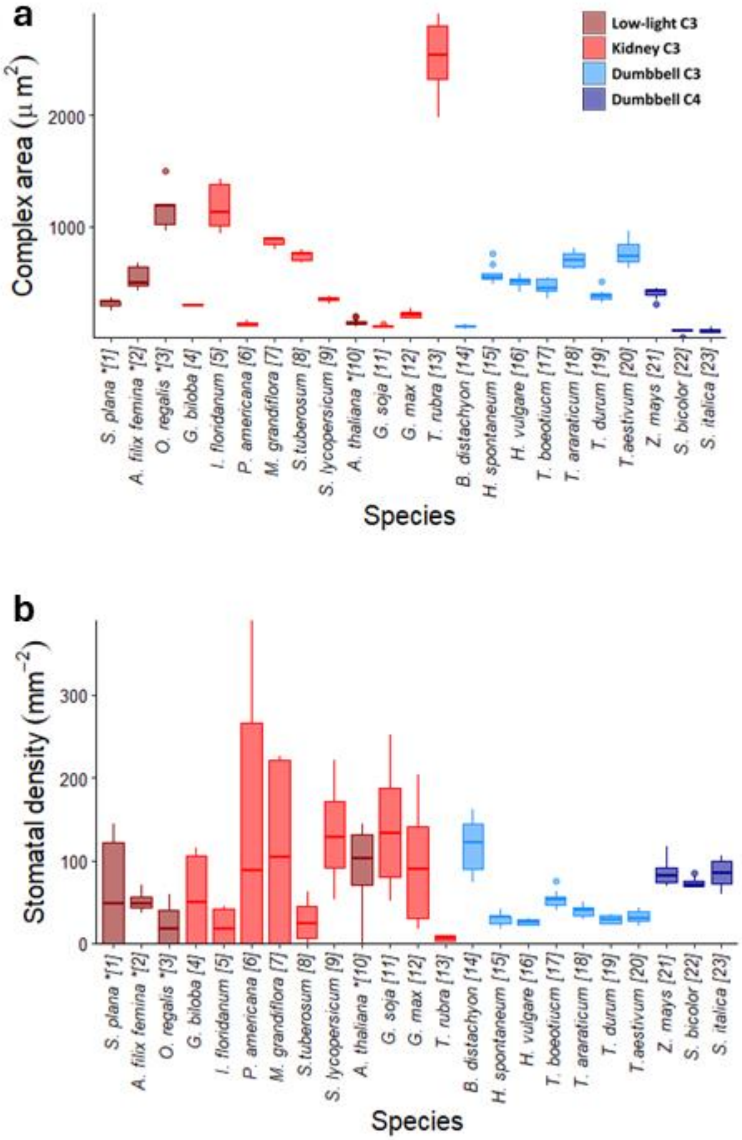

Figure S2. Stomatal sizes and densities for individual species. Stomatal complex sizes and stomatal densities for each species were calculated from the mean of abaxial and adaxial (where present) measurements. n = 3-8 plants per species. Numbers indicate species identity as in Table 1. and colours indicate species with kidney-shaped (red) and dumbbell-shaped (blue) guard cells.

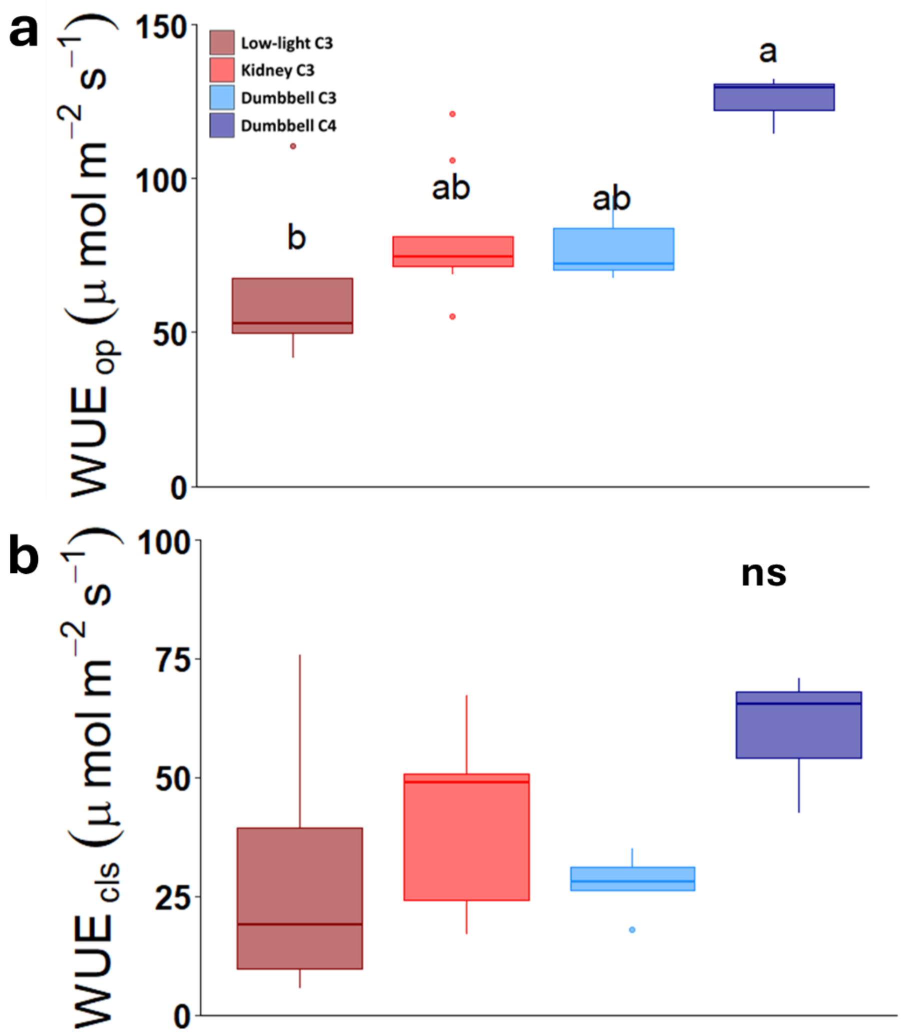

Figure S3. iWUE during opening and closure. iWUE achieved during over 30 minutes of stomatal opening (a) and closure (b). Box plots indicate median and interquartile range. Species that cannot be distinguished from each other at 0.05 confidence limit are indicated by the same letters as determined by a Kruskal-Wallis test with a Dunn test. ns = not significant across all groups. n = of 3-8 per species. Line and box colours indicate species groups: Dark red = Kidney (low light) C3; Light red = Kidney (high light) C3; Light blue = Dumbbell C3; Dark blue = Dumbbell C4.

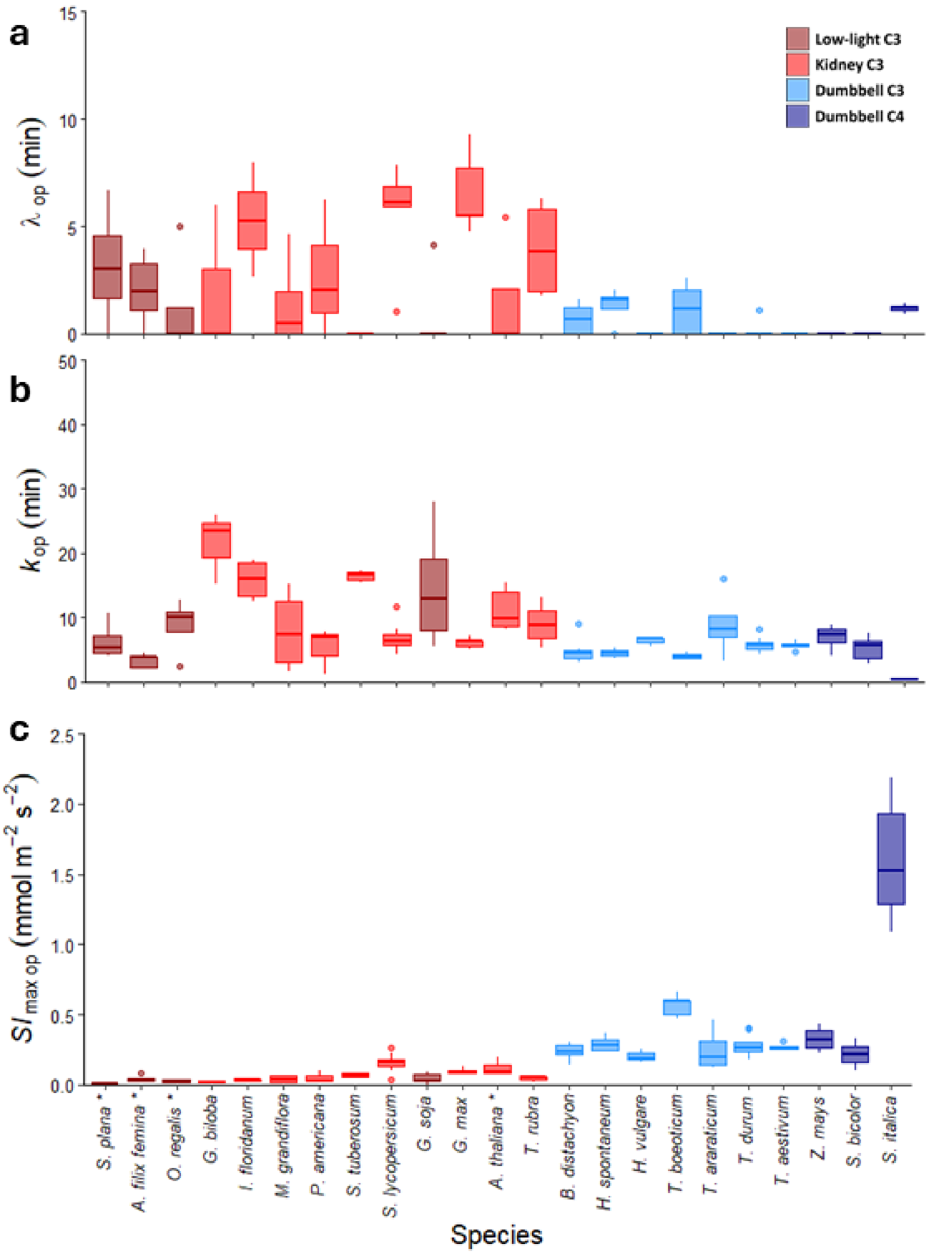

Figure S4. Stomatal opening speed parameters for individual species. Speed parameters extracted from normalised data shown in Figure 4A: a. Lag times before stomatal opening (λ_{op}); b. time to achieve 63% change in g_s during stomatal opening and closure (k_{op}); c. maximal rate of change of g_s during stomatal opening and closure ($Sl_{max\ op}$). n = 3-8 plants per species. Box plots indicate median and interquartile range. Numbers indicate species identity as in Table 1. and colours indicate species group: Kidney (low light) C3 = dark red; Kidney (high light) C3 = light red; Dumbbell C3 = light blue; Dumbbell C4 =dark blue. Individual species values plotted here were analysed in species groupings to create Figures 4b, d and f.

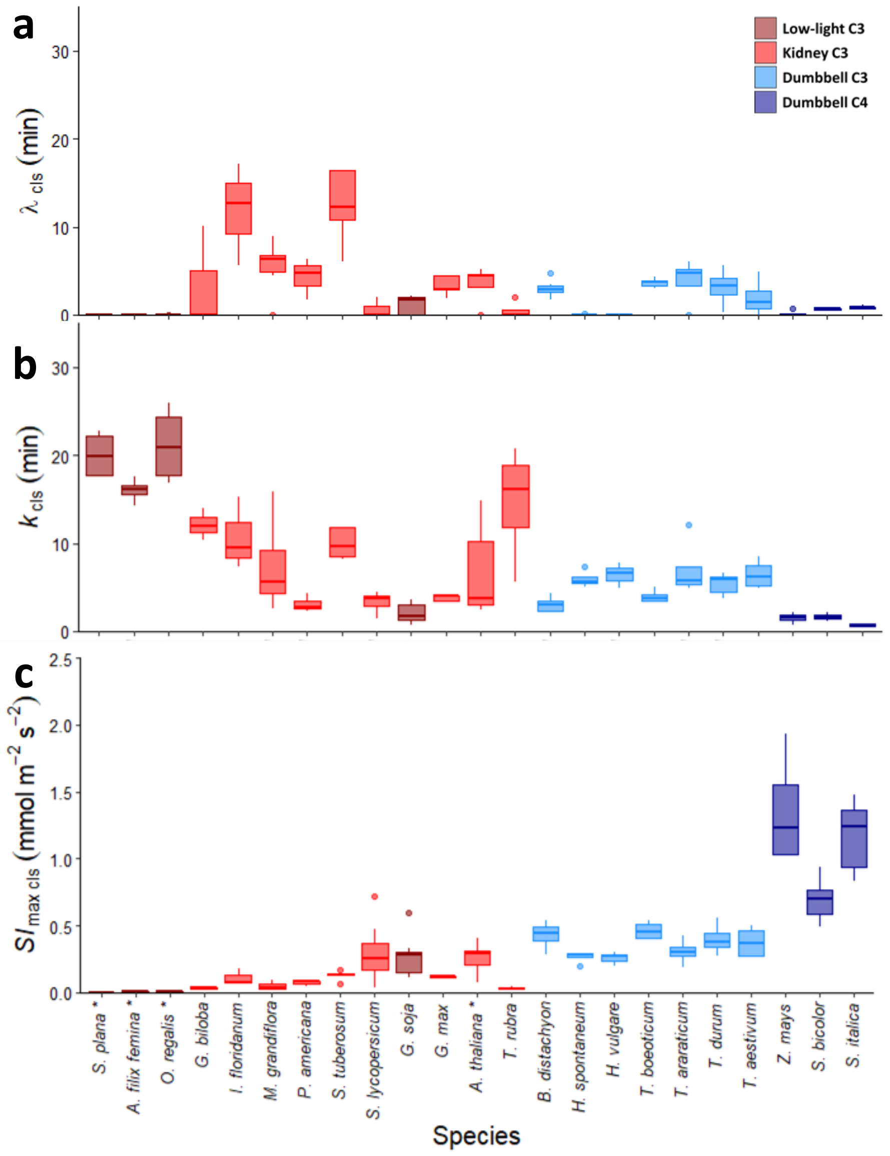

Figure S5. Stomatal closing speed parameters for individual species. Speed parameters extracted from normalised data shown in Figure 4A: a. Lag times before stomatal closing (λ_{cls}); b. time to achieve 63% change in g_s during stomatal opening and closure (k_{cls}); c. maximal rate of change of g_s during stomatal opening and closure ($SI_{max\ cls}$). n = 3-8 plants per species. Box plots indicate median and interquartile range. Numbers indicate species identity as in Table 1. and colours indicate species group: Kidney (low light) C3 = dark red; Kidney (high light) C3 = light red; Dumbbell C3 = light blue; Dumbbell C4 =dark blue. Individual species values plotted here were analysed in species groupings to create Figures 4c, e and g.

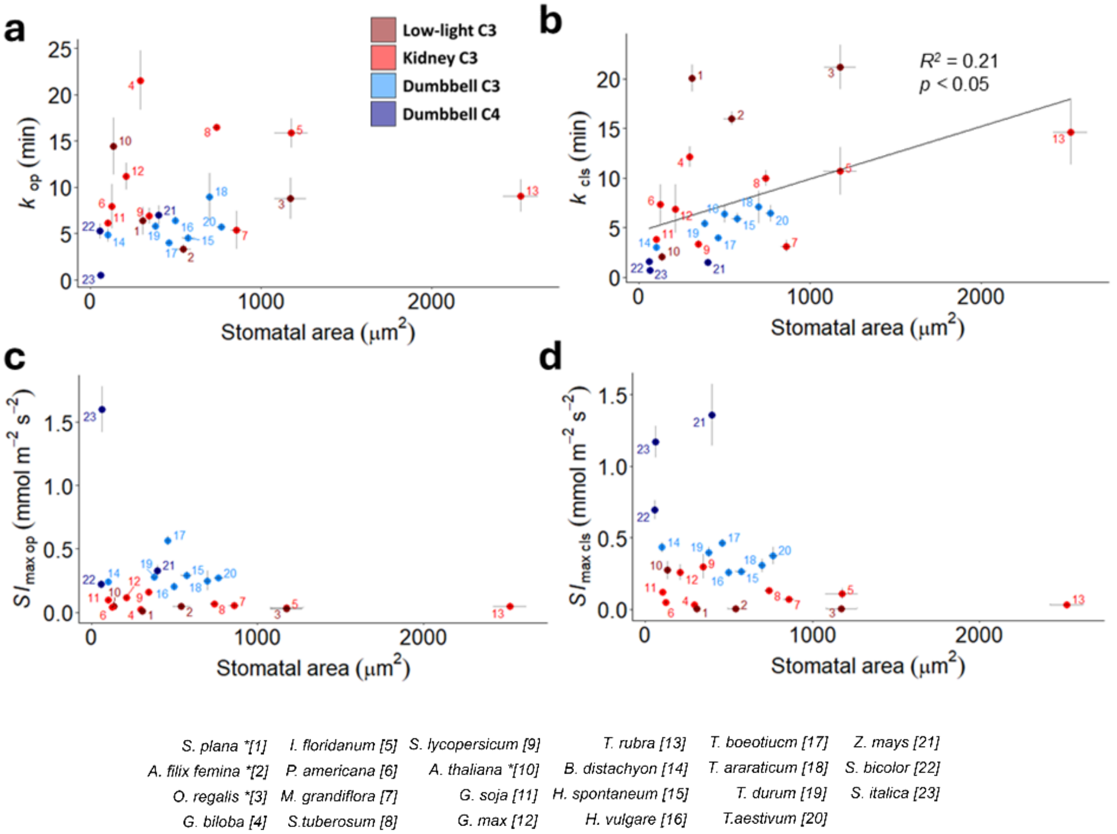

Figure S6. Lack of relationship between stomatal size and speed parameters. Correlations (Pearson correlation. Only significant correlations included) between stomatal complex areas (as shown in Figure S2) and stomatal opening and closing parameters: a. k_{op} and b. k_{cls}. c. $Sl_{max\ op}$, d. $Sl_{max\ cls}$ (as shown in Figures S3 and S4). Error bars indicate SE. Symbol colours indicate species groups: Dark red = Kidney (low light) C3; Light red = Kidney (high light) C3; Light blue = Dumbbell C3; Dark blue = Dumbbell C4. For each panel, numbered data points indicate species as shown in the figure and in Table 1.
